# Identification of retinal oligomeric, citrullinated, and other tau isoforms in early and advanced AD and relations to disease status

**DOI:** 10.1007/s00401-024-02760-8

**Published:** 2024-07-09

**Authors:** Haoshen Shi, Nazanin Mirzaei, Yosef Koronyo, Miyah R. Davis, Edward Robinson, Gila M. Braun, Ousman Jallow, Altan Rentsendorj, V. Krishnan Ramanujan, Justyna Fert-Bober, Andrei A. Kramerov, Alexander V. Ljubimov, Lon S. Schneider, Warren G. Tourtellotte, Debra Hawes, Julie A. Schneider, Keith L. Black, Rakez Kayed, Maj-Linda B. Selenica, Daniel C. Lee, Dieu-Trang Fuchs, Maya Koronyo-Hamaoui

**Affiliations:** 1 Department of Neurosurgery, Maxine Dunitz Neurosurgical Research Institute, Cedars-Sinai Medical Center, 127 S. San Vicente Blvd., A6212, Los Angeles, CA 90048 USA; 2 Department of Pathology and Laboratory Medicine, Cedars-Sinai Medical Center, Los Angeles, CA USA; 3 Smidt Heart Institute, Cedars-Sinai Medical Center, Los Angeles, CA USA; 4 Advanced Clinical Biosystems Research Institute, Cedars-Sinai Medical Center, Los Angeles, CA USA; 5 Eye Program, Board of Governors Regenerative Medicine Institute, Cedars-Sinai Medical Center, Los Angeles, CA USA; 6 Division of Applied Cell Biology and Physiology, Department of Biomedical Sciences, Cedars-Sinai Medical Center, Los Angeles, CA USA; 7 Departments of Psychiatry and the Behavioral Sciences and Neurology, Keck School of Medicine, University of Southern California, Los Angeles, CA USA; 8 Department of Pathology Program in Neuroscience, Keck School of Medicine, University of Southern California, Los Angeles, CA USA; 9 Alzheimer’s Disease Center, Rush University Medical Center, Chicago, IL USA; 10 Mitchell Center for Neurodegenerative Diseases, University of Texas Medical Branch, Galveston, TX USA; 11 Departments of Neurology, Neuroscience, and Cell Biology, University of Texas Medical Branch, Galveston, TX USA; 12 Sanders-Brown Center On Aging, Department of Neuroscience, College of Medicine, University of Kentucky, Lexington, KY USA; 13 Department of Pharmaceutical Sciences, College of Pharmacy, University of South Florida, Tampa, FL USA; 14 Department of Neurology, Cedars-Sinai Medical Center, Los Angeles, CA USA

**Keywords:** Eye, Retinal biomarkers, Tauopathy, Frontotemporal lobar dementia, Dementia with Lewy bodies, Neurodegenerative diseases

## Abstract

This study investigates various pathological tau isoforms in the retina of individuals with early and advanced Alzheimer’s disease (AD), exploring their connection with disease status. Retinal cross-sections from predefined superior-temporal and inferior-temporal subregions and corresponding brains from neuropathologically confirmed AD patients with a clinical diagnosis of either mild cognitive impairment (MCI) or dementia (*n* = 45) were compared with retinas from age- and sex-matched individuals with normal cognition (*n* = 30) and non-AD dementia (*n* = 4). Retinal tau isoforms, including tau tangles, paired helical filament of tau (PHF-tau), oligomeric-tau (Oligo-tau), hyperphosphorylated-tau (p-tau), and citrullinated-tau (Cit-tau), were stereologically analyzed by immunohistochemistry and Nanostring GeoMx digital spatial profiling, and correlated with clinical and neuropathological outcomes. Our data indicated significant increases in various AD-related pretangle tau isoforms, especially p-tau (AT8, 2.9-fold, pS396-tau, 2.6-fold), Cit-tau at arginine residue 209 (CitR_209_-tau; 4.1-fold), and Oligo-tau (T22^+^, 9.2-fold), as well as pretangle and mature tau tangle forms like MC-1-positive (1.8-fold) and PHF-tau (2.3-fold), in AD compared to control retinas. MCI retinas also exhibited substantial increases in Oligo-tau (5.2-fold), CitR_209_-tau (3.5-fold), and pS396-tau (2.2-fold). Nanostring GeoMx analysis confirmed elevated retinal p-tau at epitopes: Ser214 (2.3-fold), Ser396 (2.6-fold), Ser404 (2.4-fold), and Thr231 (1.8-fold), particularly in MCI patients. Strong associations were found between retinal tau isoforms versus brain pathology and cognitive status: a) retinal Oligo-tau vs. Braak stage, neurofibrillary tangles (NFTs), and CDR cognitive scores (*ρ* = 0.63–0.71), b) retinal PHF-tau vs. neuropil threads (NTs) and ABC scores (*ρ* = 0.69–0.71), and c) retinal pS396-tau vs. NTs, NFTs, and ABC scores (*ρ* = 0.67–0.74). Notably, retinal Oligo-tau strongly correlated with retinal Aβ_42_ and arterial Aβ_40_ forms (*r* = 0.76–0.86). Overall, this study identifies and quantifies diverse retinal tau isoforms in MCI and AD patients, underscoring their link to brain pathology and cognition. These findings advocate for further exploration of retinal tauopathy biomarkers to facilitate AD detection and monitoring via noninvasive retinal imaging.

**Supplementary Information:**

The online version contains supplementary material available at 10.1007/s00401-024-02760-8.

## Introduction

Alzheimer’s disease (AD) is the leading cause of senile dementia worldwide [1]. The pathological hallmarks of AD are characterized by the accumulation of amyloid beta-protein (Aβ) deposits and abnormal microtubulin-associated tau protein (encoded by *MAPT* gene) aggregates in the brain [14]. During the pathogenesis of AD, the tau protein undergoes post-translational modifications, such as hyperphosphorylation (p-tau) and citrullination (Cit-tau), which have an increased tendency to create toxic oligomers (Oligo-tau) [46, 68]. These oligomers can propagate to healthy neurons and spread the disease [12, 26, 32, 42, 49, 52]. As the disease progresses, pathological tau species aggregate into fibrils and paired helical filaments (PHF), ultimately forming intraneuronal neurofibrillary tangles (NFTs) [38]. Both PHF-tau and NFTs can damage cytoplasmic functions and interfere with axonal transport, leading to neuronal cell death. Cerebral Aβ accumulation occurs decades prior to the manifestation of clinical symptoms [39, 76, 77], while the subsequent increase in abnormal tau is strongly associated with neurodegeneration and disease progression [16, 32, 39]. The extended preclinical phase of AD allows for earlier and more effective interventions before severe neuronal loss occurs. Indeed, recent studies indicate enhanced efficacy of anti-amyloid immunotherapies in early AD patients [75, 83] and in individuals with a low brain tau burden, emphasizing the urgency of developing feasible, affordable, and non-invasive techniques for early screening and monitoring of AD.

The retina, an extension of the brain and the only central nervous system (CNS) organ not shielded by bone [24, 66], holds promise for revolutionizing AD screening. Current limitations in early diagnosis and monitoring of AD in the clinical settings [3, 41], make retinal imaging a potential solution due to its accessibility for noninvasive, repeated, low-cost imaging at ultra-high spatial resolution [27]. Our group and others have demonstrated the manifestation of AD pathological features in the retinas of asymptomatic individuals, those with mild cognitive impairment (MCI), and AD dementia patients, including Aβ deposits, vascular Aβ_40_ and Aβ_42_, various aberrant tau isoforms, inflammation, vascular damage, and neurodegeneration [4, 6–8, 13, 17, 20–23, 27, 31, 33–35, 43–45, 47, 50, 51, 56, 59, 63, 65, 67, 70, 71, 73, 74, 79, 87, 89]. Some pathological changes associated with AD, such as Aβ deposits, tau isoforms, vascular damage, and atrophy, were more frequently detected or had a greater impact in the superior temporal (ST) or inferior temporal (IT) retinal regions of AD patients compared to controls [7, 20, 31, 43, 44, 47, 50, 74]. In the context of tau pathology, examination of postmortem retinal tissues from AD patients detected total tau, 3- and 4-repeat tau, p-tau forms, and NFTs-like structures [20, 21, 31, 34, 43, 65, 87]. Retinal p-tau Ser202/Thr205 and Thr217 burdens correlated with Braak stage and p-tau Ser202/Thr205 burden in AD brains [34]. Additionally, the severity stages of retinal p-tau Ser202/Thr205 correlated with Aβ phases in AD brains [87]. Nevertheless, it remains unclear whether other pathological pretangle and mature tau tangle forms exist and increase in the retina of AD patients at the earliest functional impairment (MCI) and dementia stages and, moreover, correlate with brain pathology and cognitive status.

To address these questions, we investigated aberrant tau species associated with AD severity [9, 40, 57], including various pretangle forms such as p-tau, Cit-tau, and tau oligomers, as well as pretangle and mature MC-1-positive and PHF-1-positive tau tangle forms in postmortem retinal tissues from a cohort of individuals with MCI (due to AD), AD dementia, as compared to normal cognition (NC) and non-AD dementia (D-NAD) controls. Since previous studies suggested that AD-related pathological changes were often detected in the superior or the ST/IT retina of AD patients, we focused our examination of tau isoforms in these retinal regions. Our analyses indicate that most abnormal tau forms, predominantly pretangle pathology, significantly increase in the retina of MCI and AD patients and generally correlate with the respective brain pathology. The results of this study may offer insights into the specific pathological tau isoforms that can serve as future retinal biomarkers for AD detection and assessment of disease progression.

## Methods

### Postmortem eyes from human donors

Human eye and brain tissues were collected from donor patients with premortem clinical diagnoses of MCI and AD dementia (and confirmed postmortem AD neuropathology), along with age- and sex-matched NC controls (total *n* = 75 subjects). These tissues were primarily obtained from the Alzheimer’s Disease Research Center (ADRC) Neuropathology Core in the Department of Pathology (IRB protocol HS-042071) at the Keck School of Medicine, University of Southern California (USC, Los Angeles, CA). Additional eyes were obtained from the National Disease Research Interchange (NDRI, Philadelphia, PA) under the approved Cedars-Sinai Medical Center IRB protocol Pro00019393. Both USC-ADRC and NDRI maintain human tissue collection protocols that are approved by their managerial committees and subject to oversight by the National Institutes of Health. Histological studies at Cedars-Sinai Medical Center were performed under IRB protocols Pro00053412 and Pro00019393. Demographic, clinical, and neuropathological information on human donors are detailed in Table 1.

**Table 1:** Demographic and neuropathological data on human donors in this study

		NC	MCI	AD	*F*	*P*
*N* = 75		30 16F (53%), 14 M	11 7F (64%), 4 M	34 21F (62%), 13 M	–	–
Age at death (years)		81.43 ± 9.82	89.55 ± 6.12	83.88 ± 12.67	2.25	0.11
Race		25W (83%) 3H, 2B	9W (82%) 1H, 1B	24W (71%) 5A, 4H, 1B	–	–
PMI (hours)		28 7.48 ± 3.55	11 8.18 ± 4.90	34 8.52 ± 4.20	0.50	0.61
MMSE score		15 28.7 ± 2.2	9 20.1 ± 7.0	24 12.5 ± 8.0	21.82	** < 0.0001**
CDR score		10 0.4 ± 0.69	11 2.18 ± 1.05	31 2.45 ± 0.88	20.98	**0.0004**
Brain neuropathology (*N* = 56)	Braak stage (%)	0 (18%)	0 (9%)	0 (0%)	26.82	**0.0001**
		I–II (46%)	I–II (18%)	I–II (0%)		
		III–IV (27%)	III–IV (27%)	III–IV (24%)		
		V (9%)	V–VI (46%)	V–VI (76%)		
	ABC^b^	1.45 ± 0.83	2.26 ± 0.57	2.77 ± 0.31	28.95	** < 0.0001**
	Aβ plaque^a^	1.04 ± 1.11	2.04 ± 0.94	2.53 ± 0.94	9.69	**0.0003**
	NFTs^a^	0.42 ± 0.47	1.95 ± 1.05	2.39 ± 0.91	20.90	** < 0.0001**
	NTs^a^	0.40 ± 0.81	1.10 ± 0.75	1.83 ± 1.15	8.76	**0.0005**
	Atrophy^a^	0.80 ± 0.98	1.11 ± 0.96	1.81 ± 1.10	4.63	**0.014**

List of human donors’ retinas (total *n* = 75 subjects) included in this study. Corresponding brains with full neuropathological assessments were available for 56 human donors (NC = 11; MCI = 11; AD = 34). Mean ABC scores were determined as follows: A, Aβ plaque score modified from Thal; B, NFT stage modified from Braak; C, neuritic plaque score modified from CERAD. Group values are presented as mean ± standard deviation. *F* and *P*-values were determined using one-way analysis of variance with Tukey's multiple comparisons test. *P* values presented in bold type demonstrate significance.

*Aβ* amyloid beta-protein, *AD* Alzheimer's disease, *A* Asian, *B* Black, *CDR* Clinical Dementia Rating, *CERAD* Consortium to Establish a Registry for Alzheimer's Disease, *NC* normal cognition controls, *F* female, *H* Hispanic, *M* male, *MCI* mild cognitive impairment, *MMSE* Mini-Mental State Examination, *NFTs* neurofibrillary tangles, *NTs* neuropil threads, *PMI* post mortem interval, *SD* standard deviation, *W* White

^a^Severity score 0*–*5

^b^Average score

The available brain and retinal tissues from individual donors for each analysis are specified in Suppl. Table 1. For the histopathological analysis, the human cohort consisted of AD dementia (*n* = 34), MCI (*n* = 11), and NC controls (*n* = 30). Additionally, retinas from non-AD dementia patient donors (*n* = 4) were included in the histological analyses. These consisted of a patient with dementia Lewy bodies (DLB) with sparse brain AT8^+^-tau and NFTs, a patient with frontotemporal lobar degeneration (FTLD) with amyotrophic lateral sclerosis C9orf72 (ALS) with no brain tauopathy and sparse NFTs, a patient with FTLD/Pick’s disease with brain tauopathy, positive PHF-tau, and 3-repeat isoform of tau, but no NFTs, and a patient with FTLD/progressive supranuclear palsy (PSP) with brain tauopathy and positive PHF-tau, but no NFTs. For the NanoString GeoMx analysis, the cohort consisted of AD dementia (Retina: *n* = 9; Brain: *n* = 4), MCI (Retina: *n* = 6; Brain: *n* = 4), and NC (Retina: *n* = 9; Brain: *n* = 5). Patients’ identities were protected by de-identifying all tissue samples in a manner not allowing them to be traced back to tissue donors.

### Clinical and neuropathological assessments

The ADRC provided the clinical and neuropathological reports on the patients’ neurological examinations, neuropsychological and cognitive tests, family history, and medication lists as collected in the ADRC system using the Unified Data Set (UDS) [11]. The NDRI provided the medical history of additional patients. Subjects with a history of diabetic retinopathy, macular degeneration, or glaucoma were excluded in this study. Most cognitive evaluations had been performed annually, and, in most cases, less than one year prior to death. Cognitive testing scores from evaluations made closest to the patient’s death were used for this analysis. Two global indicators of cognitive status were used for clinical assessment: the Clinical Dementia Rating (CDR scores: 0 = normal; 0.5 = very mild impairment; 1 = mild dementia; 2 = moderate dementia; or 3 = severe dementia) [60] and the Mini-Mental State Examination (MMSE scores: 24–30 = NC; 20–23 = MCI; 10–19 = moderate dementia; or 9 ≥ severe dementia) [25]. In this study, the composition of the clinical diagnostic group (AD, MCI, or CN) was determined by source clinicians based on findings of a comprehensive battery of tests including neurological examinations, neuropsychological evaluations, and the aforementioned cognitive tests. Specifically, the definition of MCI due to AD was assigned to patients who had the antemortem clinical diagnosis of MCI (based on the comprehensive battery of behavioral and cognitive tests) that was caused by AD. Meaning, these patients had a postmortem confirmation of AD neuropathology (according to the ADNC—Alzheimer's disease neuropathological change guidelines), and no evidence of other diseases, such as Lewy body dementia, Parkinson's disease, FTD/FTLD (PSP or Pick’s disease), or any cognitive impairment due to stroke or small vessel disease.

To obtain a final diagnosis based on the neuropathological reports, we used the modified Consortium to Establish a Registry for Alzheimer's Disease (CERAD) [55, 69], as outlined in the National Institute on Aging (NIA)/Regan protocols with revision by the NIA and Alzheimer’s Association [37]. The Aβ burden (measured as diffuse, immature, or mature plaques), amyloid angiopathy, neuritic plaques, NFTs, neuropil threads (NTs), granulovacuolar degeneration, Lewy bodies, Hirano bodies, Pick bodies, balloon cells, neuronal loss, microvascular changes, and gliosis pathology were assessed in multiple brain areas, including the hippocampus (particularly the Cornu ammonis CA1, at the level of the thalamic lateral geniculate body), entorhinal cortex, superior frontal gyrus of the frontal lobe, superior temporal gyrus of the temporal lobe, superior parietal lobule of the parietal lobe, primary visual cortex (Brodmann Area-17), and visual association (Area-18) of the occipital lobe. In all cases, uniform brain sampling was done by a neuropathologist.

Cerebral amyloid plaques, NFTs, and NTs were evaluated using anti–β-amyloid mAb clone 4G8 immunostaining, Thioflavin-S (ThioS) histochemical stain, and Gallyas silver stain in formalin-fixed, paraffin-embedded tissue sections. The ADRC neuropathologists determined the severity scores based on semi-quantitative observations. The scale for Aβ/neuritic plaques was determined by 4G8- and/or Thioflavin-S-positive and/or Gallyas silver-positive plaques measured per 1 mm^2^ brain area (0 = none; 1 = sparse [≤ 5 plaques]; 3 = moderate [6–20 plaques]; 5 = abundant/frequent [21–30 plaques or greater]; or N/A = not applicable), as previously described [58]; NACC NP Guidebook, Version 10, January 2014: https://naccdata.org/data-collection/forms-documentation/np-10. Brain NFT or NT severity scoring system was derived from observed burden of these AD neuropathologic changes detected by Gallyas silver and/or Thioflavin-S staining [57, 58, 82] and measured per 1 mm^2^ brain area. The assigned NFT or NT scores are as follows: 0 = none; 1 = sparse (mild burden); 3 = moderate (intermediate burden); or 5 = frequent (severe burden). In both histochemical and immunohistochemical staining, each anatomic area of interest is assessed for the relevant pathology using the 20× objective (200× high power magnification), and representative fields are graded using a semiquantitative scale as detailed above. Validation of AD neuropathic change (ADNP), especially NTs, is performed using the 40× objectives (400× high power magnification); an average of two readings was assigned to each individual patient.

A final diagnosis included AD neuropathological change using an “ABC” score derived from three separate 4-point scales. We used the modified Aβ plaque Thal score (A0 = no Aβ or amyloid plaques; A1 = Thal phase 1 or 2; A2 = Thal phase 3; or A3 = Thal phase 4 or 5) [80]. For the NFT stage, the modified Braak staging for silver-based histochemistry or p-tau IHC was used (B0 = no NFTs; B1 = Braak stage I or II; B2 = Braak stage III or IV; or B3 = Braak stage V or VI) [15]. For the neuritic plaques, we used the modified CERAD score (C0 = no neuritic plaques; C1 = CERAD score sparse; C2 = CERAD score moderate; or C3 = CERAD score frequent) [55]. Neuronal loss, gliosis, granulovacuolar degeneration, Hirano bodies, Lewy bodies, Pick bodies, and balloon cells were all evaluated (0 = absent or 1 = present) in multiple brain areas by staining tissues with hematoxylin and eosin (H&E). Brain atrophy was evaluated (0 = none; 1 = mild; 3 = moderate; 5 = severe; or 9 = not applicable).

### Processing of eye and brain tissues

Donor eyes were collected within an average of 8 h after the time of death and were subjected to the following preservation methods: (1) preserved in Optisol-GS media (Bausch & Lomb, 50006-OPT) and stored at 4 °C for less than 24 h; (2) fresh-frozen (snap-frozen) and stored at − 80 °C; or (3) punctured once and fixed in 10% neutral buffered formalin (NBF) or 4% paraformaldehyde (PFA) and stored at 4 °C. Regardless of the source of the human donor eye (USC-ADRC or NDRI), the same tissue collection and processing methods were applied. Fresh brain tissues from the same donors were snap-frozen and stored at − 80 °C. Portions of fresh-frozen brain tissues were fixed in 4% PFA for 16 h following dehydration in 30% sucrose in 1× phosphate-buffered saline (PBS). Alternatively, brain tissues were fixed in 4% PFA and then embedded in paraffin using standard techniques. Next, brains were sectioned at 30 μm thickness and mounted on microscope slides coated with 3-aminopropyltriethoxysilane (APES, Sigma A3648).

### Preparation of retinal strips

Eyes that were fixed in 10% NBF or 4% PFA were dissected as previously described [43, 44, 74]. Briefly, after careful dissection and thorough cleaning of the vitreous humor, flat mount strips (~ 2 mm wide) extending diagonally from the optic disc (OD) to the ora serrata (~ 20 to 25 mm long) were prepared to create 4 strips (Superior Temporal ST, Inferior Temporal IT, Inferior Nasal IN, and Superior Nasal SN). The flat mount-derived strips were then paraffinized using standard techniques and embedded in paraffin after flip-rotating 90° horizontally. Next, the retinal strips were sectioned (7–10 µm thick) and mounted on microscope slides coated with APES. This sample preparation technique allowed for extensive and consistent access to retinal quadrants, layers, and pathological subregions.

### Immunofluorescent staining

Brain and retinal sections were deparaffinized with 100% xylene twice (10 min each), rehydrated with decreasing concentrations of ethanol (100% to 70%), and washed with distilled water followed by PBS. Following deparaffinization, tissue sections were treated with target retrieval solution (pH 6.1; S1699, DAKO) at 98 °C for 1 h and then washed with PBS. Next, tissues were incubated in blocking buffer (Dako #X0909) supplemented with 0.1% Triton X-100 (Sigma, T8787) for 1 h at room temperature (RT), followed by overnight primary antibody (Ab) incubation at 4 °C (Abs information provided in Suppl. Table 2). Sections were then washed three times with PBS and incubated with secondary Abs against each species (1:200, Suppl. Table 2) for 1 h at RT. After rinsing with PBS three times, sections were mounted with Prolong Gold antifade reagent with DAPI (Thermo Fisher #P36935).

### Peroxidase-based immunostaining

After deparaffinization and antigen retrieval treatment, the tissues were treated with 70% formic acid (ACROS) for 10 min at room temperature, followed by 3% H_2_O_2_ for 10 min using two staining protocols: (1) Vectastain Elite ABC HRP kit (Vector, PK-6102, Peroxidase Mouse IgG) according to the manufacturer’s instructions, or (2) all Dako reagents protocol as follows: after formic acid treatment, the tissues were washed with wash buffer (Dako S3006) supplemented with 0.1% Triton X-100 (Sigma, T8787) for 1 h, then treated with H_2_O_2_ and rinsed with wash buffer. Primary Ab (Suppl. Table 2) was diluted with background reducing components (Dako S3022) and incubated with the tissues overnight at 4 °C. Tissues were rinsed thrice with wash buffer on a shaker and incubated for 30 min at 37 °C with secondary Ab (goat anti-mouse or anti-rabbit HRP conjugated, DAKO Envision K4001 and K4003, respectively), then rinsed again thrice with wash buffer on a shaker. For both protocols, diaminobenzidine (DAB) substrate was used (DAKO K3468). Counterstaining with hematoxylin was performed followed by mounting with Faramount aqueous mounting medium (Dako, S3025). Routine controls were processed using identical protocols while omitting the primary antibodies to assess nonspecific labeling.

### Bielschowsky’s silver staining

Fixed brain sections and retinal cross-sections were deparaffinized and then processed for silver staining using the Hito Bielshowsky OptimStainTM Kit (Hito, #HTNKS1126). The protocol was optimized for our samples. Sections were first incubated with solution-1 for 22.5 min at 4 °C in a dark humidity chamber. Meanwhile, the Developer solution was prepared by mixing 1 ml of solution-1 with 600 µl of solution-2 (add 10 µl solution-2 for 17–20 times until solution is clear). A portion of the final solution was set aside (1 ml) and labeled as Developer solution. After incubation, each section was rinsed three times with distilled water, followed by the addition of the prepared mixture onto sections and then incubated in a dark humidity chamber at 4 °C for 22.5 min. The slides were then quickly rinsed in distilled water. Double distilled water (50 ml) and solution-2 (200 μl) were added to a coplin jar and mixed well. Slides were then placed in the coplin jar, once incubation was completed, until the developing step. Solution-3 (60 μl) and Developer solution (1 ml) were combined in an Eppendorf tube. The new solution was immediately applied to the sections until fully covered. Sections were left covered in a humidity chamber, with the degree of color change routinely checked every 30 s to 1 min. After 4 min, sections were examined under a light microscope to assess the staining level. The sections should turn brown, taking approximately 7.5–8 min to achieve full staining (times may vary). Once the tissue appeared golden brown, the sections were placed in a coplin jar for 1 min. Solution-4 was added to a 12-ml staining jar (provided by the kit), and the slides were added to the staining jar for 3 min at room temperature. Slides were then rinsed in double distilled water two times for 2 min each and dehydrated in 50% EtOH, 75% EtOH, 95% EtOH, and 100% EtOH with two changes in each step, and 3–5 min during each change. The slides were cleared in xylene two times for 4 min each. A resinous-based mounting media and coverslips were applied to each slide and allowed to dry for brightfield microscopy.

### Microscopy

Fluorescence and brightfield images were acquired using a Carl Zeiss Axio Imager Z1 fluorescence microscope (with motorized Z-drive) equipped with ApoTome, an AxioCam HRc, and AxioCam MRm monochrome cameras (version 3.0; at a resolution of 1388 × 1040 pixels, 6.45 µm × 6.45 µm pixel size, and a dynamic range of > 1:2200, which delivers low-noise images due to a Peltier-cooled sensor) with ZEN 2.6 blue edition software (Carl Zeiss MicroImaging, Inc.). Multi-channel image acquisition was used to create images with multiple channels. Images were consistently captured at the same focal planes with identical exposure time.

### Stereological quantification

Images were captured at 20× or 40× objectives, at a respective resolution of 0.25 µm. Approximately, 20 images were obtained from each retina. The acquired images were converted to grayscale and standardized to baseline using a histogram-based threshold in the Fiji ImageJ (NIH) software program (version 1.53c). For each biomarker, the total area of immunoreactivity was determined using the same threshold percentage from the baseline in ImageJ (with the same percentage threshold setting for all diagnostic groups). The images were then subjected to particle analysis to determine the immunoreactive (IR) area and/or area fraction (%).

### NanoString GeoMx^®^ Digital Spatial Profiling of total tau and phosphorylated tau (p-tau)

Formalin fixed paraffin embedded human brain (A-9; frontal lobe) and retinal cross-sections from ST and IT regions (spanning from the optic disc to the ora serrata) were deparaffinized and then treated with antigen retrieval solution, 70% formic acid (ACROS) and blocking solution (supplemented with 0.1% Triton X-100; Sigma, T8787). After this, the sections were subjected to overnight incubation with primary Abs (for morphology markers) at 4 °C. Secondary Abs (Cy2 and Cy3) were added the following day and incubated for 1.5 h at RT.

Morphological markers in IHC were utilized to select regions of interest (ROI). For ROI selection, whole slides were stained using three morphology markers: 1. Tissue marker—Aβ Ab (MOAB-2; NBP2-13075; Novus; dilution 1:500) recognizes unaggregated, oligomeric, and fibrillar forms of Aβ_42_ and unaggregated Aβ_40_ but does not detect APP. 2. Immune cell marker—Iba1/AIF1 Ab recognizes microglial/macrophage (20A12.1; 970896; EMD Milipore; dilution 1:300). 3. DNA marker—Syto 13. For all subject groups (AD, MCI, and NC), the same ROI dimensions were selected from the retinal Central (C), Mid (M), and Far (F) subregions, with a total of three ROIs per retinal cross section. Similarly, the same ROI dimensions were selected from each brain section, with a total of two ROIs per brain section.

For the tau module, a panel of digital spatial profiler (DSP) barcoded antibodies were used in GeoMx Protein assays. The DSP barcode was conjugated to the Ab with a photocleavable linker. The Abs were against total tau and p-tau (S214; T231; S199; S396; S404).

Following staining, the slides were imaged and profiled using GeoMx^®^ DSP and tissues were exposed to UV light in the selected ROI, causing the cleavage of the DSP barcode from the Ab. The protein expression level was then collected and quantified directly by digitally counting the released barcodes using the nCounter^®^ Analysis System. Raw data was analyzed with the DSP Data Analysis Suite (DSPDA), after which the data was normalized to the housekeeping protein GAPDH, which was detected with the Tau module in the same experiment.

## Statistical analysis

GraphPad Prism version 10.2.3 (GraphPad Software) was used for the analyses. Three or more group comparisons were analyzed using one-way ANOVA followed by Tukey’s or Sidak’s multiple comparison post-tests. Two-group comparisons were analyzed using a two-tailed unpaired Student *t* test. In two-way ANOVA analyses, the *P*d (diagnosis), *P*r (C/M/F regions), and *P*i (interactions) were presented. Spearman's rank correlation coefficient analyses (non-parametric) were conducted to determine the statistical association between retinal tau isoforms and brain pathology parameters and cognitive scores. Pearson’s *r* correlation coefficient analyses (parametric) were conducted to determine the statistical association between retinal tau isoforms and retinal Aβ forms. Pair-wise Pearson’s (*r*_*P*_) or Spearman's (*ρ* or *r*_*S*_) coefficient were used to indicate the direction and strength of the linear relationship between two variables. Required sample sizes for two group (differential mean) comparisons were calculated using the nQUERY *t*-test model, assuming a two-sided *α* level of 0.05, 80% power, and unequal variances, with the means and common standard deviations for the different parameters. Results are expressed as means ± SDs in tables and median, lower and upper quartile in violin plots. Degrees of significance are presented as: *P < 0.05, **P < 0.01, ***P < 0.001, and ****P < 0.0001.

### Data availability

Most data generated or analyzed for this study are included in this published manuscript and supplementary online material. Additional data will be made available by the contact PI upon reasonable request.

## Results

**Fig. 1:**
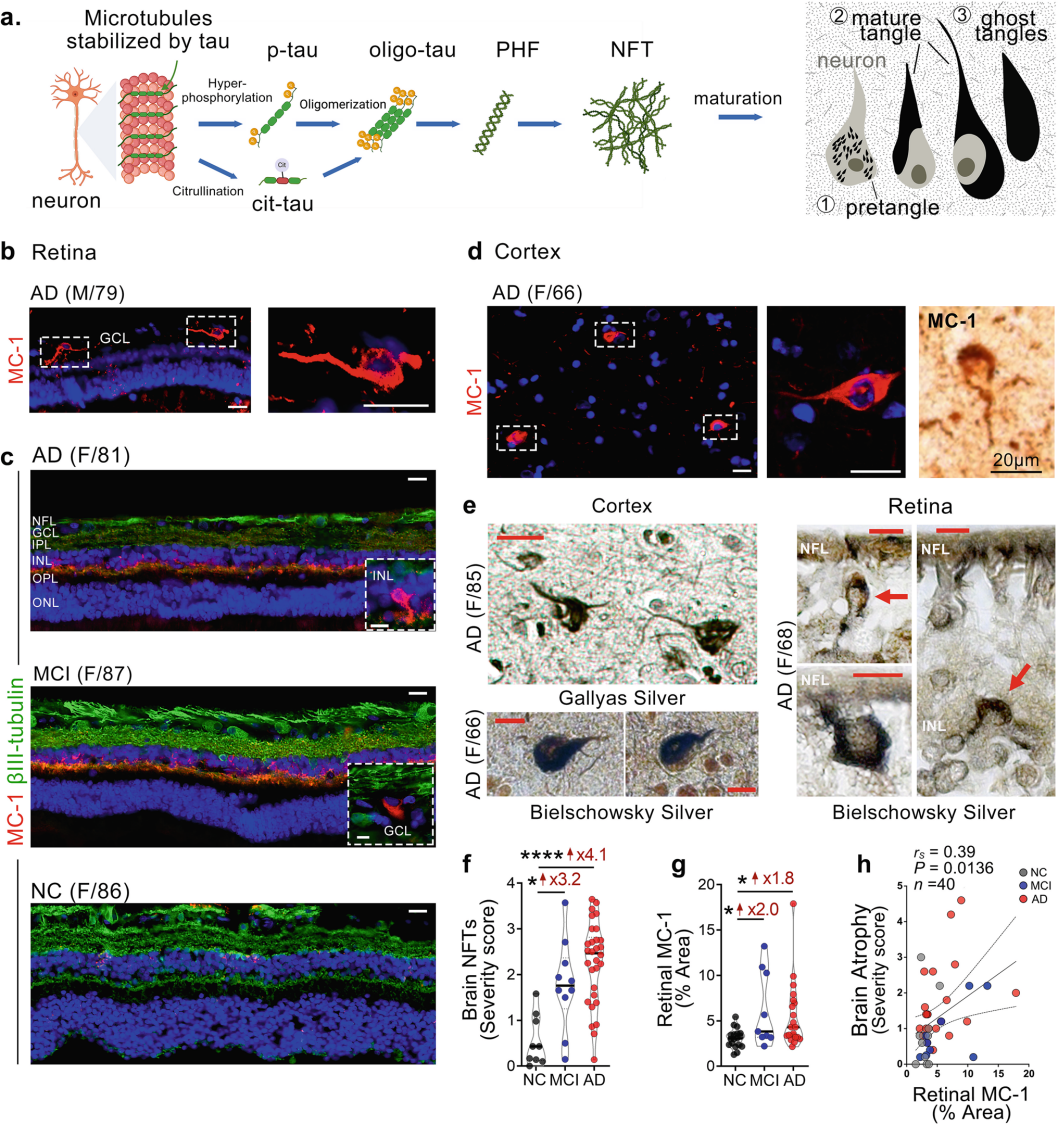
MC-1-positive tau tangles in the retina of MCI and AD patients. **a** Illustration of the various abnormal tau isoforms. **b**, **c** Representative images of immunofluorescent staining for MC-1^+^ tau tangles (red), neuronal marker βIII-tubulin (green), and DAPI (blue) in retinal cross-sections from individuals with normal cognition (NC), as well as patients with MCI and AD dementia. Scale bars 20 µm. Additional inserts of high-magnification images show intra-neuronal MC-1^+^ tau tangles in the MCI and AD retinas (scale bars 10 µm). **d** Representative images of cortical (A9) stained for MC1^+^ tau tangles using immunofluorescence (red) or peroxidase-based DAB (brown) labeling. Scale bars 20 µm. **e** Gallyas silver staining (Scale bars 20 µm) and/or Bielschowsky silver staining (Scale bars 10 µm) in cortical and retinal sections from AD patients. **f** Quantitative assessment of brain NFT severity scores in patients with MCI, AD, and NC controls (*n* = 49 in total). **g** Quantitative analysis of the percentage of retinal MC-1^+^ immunoreactive area (*n* = 18 NC, *n* = 9 MCI, and *n* = 21 AD). **h** Spearman's rank correlation coefficient analysis between retinal MC-1% area and brain atrophy severity score. Individual data points (circles) and median, lower and upper quartile are shown in violin plots. *P < 0.05, ****P < 0.0001, by one-way ANOVA with Sidak’s post hoc multiple comparison tests. Fold changes are shown in red. F, female; M, male; age (in years); *n*, sample size. Illustrations created with Biorender.com

To investigate the burden and spatiotemporal distribution of AD-related abnormal tau isoforms (Fig. 1a) in the retina, we prepared retinal cross-sections from the ST and IT regions in a cohort of patients with a premortem diagnosis of AD dementia (*n* = 34, mean age 83.88 ± 12.67 years, 21 females/13 males) or MCI (due to AD; *n* = 11, mean age 89.55 ± 6.12 years, 7 females/4 males), with postmortem neuropathological confirmation of AD, along with individuals with NC (*n* = 30, mean age 81.43 ± 9.82 years, 16 females/14 males). No significant differences were found in age, sex, or post-mortem interval (PMI) among the three diagnostic groups. Demographic information, brain neuropathology, and retinal tauopathy histological quantifications are detailed in Tables 1 and 2. The correlations of various retinal tau isoforms with brain neuropathology and cognitive scores are summarized in Table 3. As an additional control group for AD dementia, we also analyzed several tau isoforms in postmortem retinas of patients with non-AD dementia (*n* = 4), including DLB, FTLD with ALS, FTLD-tauopathy with Pick’s disease, and FTLD-tauopathy with PSP (see Methods above and Suppl. Table 1).

**Table 2:** Retinal tauopathy quantification data on human donors in this study

	*n*	NC	*n*	MCI	*n*	AD	*F*	*P*
pS202/T205-tau (AT8)^b^	18	1.35 ± 1.44	8	4.68 ± 4.78	21	3.94 ± 3.75	4.16	**0.0222**
pS212/T214-tau (AT100)^a^	9	13.69 ± 6.51	4	18.08 ± 9.63	6	21.04 ± 5.43	2.10	0.1545
pS396-tau (pS396)^a^	15	0.86 ± 0.46	9	1.92 ± 0.91	25	2.22 ± 1.43	6.93	**0.0023**
CitR_209_-tau^b^	18	3.78 ± 2.93	8	13.29 ± 10.86	21	15.65 ± 9.27	11.71	** < 0.0001**
Oligo-tau (T22)^a^	19	0.35 ± 0.25	10	1.80 ± 0.87	21	3.18 ± 1.84	24.99	** < 0.0001**
PHF-tau (PHF-1)^b^	9	392.30 ± 155.10	5	383.50 ± 188.50	10	914.00 ± 234.40	20.35	** < 0.0001**
Tau tangles (MC-1)^a^	18	3.06 ± 1.06	9	6.20 ± 4.12	21	5.49 ± 3.61	4.42	**0.0178**

Retinal tauopathy data in the same cohort as Table 1. Group values are presented as mean ± standard deviation. *F* and *P* values were determined using one-way analysis of variance with Tukey's multiple comparisons post-test. *P* values presented in bold type demonstrate significance

*AD* Alzheimer's disease, *MCI* mild cognitive impairment, *NC* normal cognition controls, *SD* standard deviation

^a^%Area for retinal MC-1, T22, AT100, and pS396 tau markers

^b^Total IR Area for retinal AT8, CitR209, and PHF-1 tau markers

**Table 3:** Spearman’s correlation analysis between retinal tauopathy markers, brain neuropathology and cognitive decline

Brain	Core AD pathology			Non-specific tissue injury	Vascular co-morbidity	AD staging		Cognition	
Retina Tauopathy (Antibody)	Aβ-P	NFT	NT	Atrophy	CAA	Braak	ABC	CDR	MMSE
pS202/T205-tau (AT8)	0.26 (*n* = 34)	0.34* (*n* = 34)	0.034 (*n* = 34)	0.026 (*n* = 34)	0.37* (*n* = 34)	0.22 (*n* = 34)	0.37* (*n* = 34)	0.53** (*n* = 33)	− 0.38* (*n* = 29)
pS212/T214-tau (AT100)	0.34 (*n* = 13)	0.65* (*n* = 13)	0.15 (*n* = 13)	0.26 (*n* = 13)	− 0.22 (*n* = 13)	0.53 (*n* = 13)	0.35 (*n* = 13)	0.20 (*n* = 11)	− 0.43 (*n* = 13)
pS396-tau (pS396)	0.61**** (*n* = 41)	0.72**** (*n* = 41)	0.74**** (*n* = 41)	0.23 (*n* = 41)	0.14 (*n* = 40)	0.58**** (*n* = 41)	0.67**** (*n* = 41)	0.34* (*n* = 39)	− 0.28 (*n* = 34)
CitR_209_-tau	0.21 (*n* = 35)	0.19 (*n* = 35)	− 0.05 (*n* = 35)	0.003 (*n* = 35)	0.22 (*n* = 35)	0.18 (*n* = 35)	0.17 (*n* = 35)	0.35* (*n* = 34)	− 0.49** (*n* = 30)
Oligo-tau (T22)	0.47** (*n* = 39)	0.66**** (*n* = 39)	0.53*** (*n* = 39)	0.21 (*n* = 39)	0.69**** (*n* = 38)	0.71**** (*n* = 39)	0.61**** (*n* = 39)	0.63**** (*n* = 38)	− 0.57*** (*n* = 31)
PHF-tau (PHF-1)	0.29 (*n* = 18)	0.51* (*n* = 18)	0.71** (*n* = 18)	0.54* (*n* = 18)	0.50* (*n* = 17)	0.53* (*n* = 18)	0.69** (*n* = 18)	0.25 (*n* = 17)	− 0.22 (*n* = 15)
Tau Tangles (MC-1)	0.083 (*n* = 40)	0.33* (*n* = 40)	0.35* (*n* = 40)	0.39* (*n* = 40)	0.33* (*n* = 39)	0.25 (*n* = 40)	0.24 (*n* = 40)	0.20 (*n* = 37)	− 0.27 (*n* = 32)

Correlations between retinal tauopathy markers vs. brain neuropathology and cognitive scores. Antibody name is noted in parathesis following each retinal tauopathy marker. Mean ABC scores were determined as follows: A, Aβ plaque score modified from Thal; B, NFT stage modified from Braak; C, neuritic plaque score modified from CERAD

Pair-wise Spearman’s rank coefficient correlation analyses showing the strength of the association (r values), sample size number in parenthesis (*n*), and statistical significance as follows: *P < 0.05, **P < 0.01, ***P < 0.001, ****P < 0.0001. *P* values are not shown for non-significant associations

*Aβ* amyloid beta-protein, *AD* Alzheimer's disease, *CAA* cerebral amyloid angiopathy, *CDR* Clinical Dementia Rating, *MMSE* Mini-Mental State Examination, *NFTs* neurofibrillary tangles, *NT* neuropil thread

### Increased MC-1-positive tau tangles in the MCI and AD retina.

To explore the presence and burden of tau tangles in the AD retina, we utilized anti-MC-1 immunostaining and Bielschowsky silver staining in retinal cross-sections from a subset of donors with MCI (*n* = 9, mean age 89.7 ± 5.1 years, 5 females/4 males), AD (*n* = 21, mean age 86.1 ± 8.4 years, 11 females/10 males), and NC controls (*n* = 18, mean age 84.5 ± 9.3 years, 9 females/9 males), comparing them to brain NFTs (Fig. 1, extended data in Suppl. Fig. 1). Specifically, we conducted IHC staining with the MC-1 monoclonal antibody, a tau tangle conformational- and sequence-specific antibody, which identifies the amino acid (aa) 7‐9 and 312‐322 tau epitopes [40]. MC-1 primarily recognizes pre-tangles and mature tangles [57]. We mostly found pretangles and diffuse-type MC-1^+^ signal in the human MCI and AD retina. Occasionally, we identified retinal MC-1^+^ mature tau tangles, indicative of the paperclip folding of tau (Fig. 1b, c), with similar structures to NFTs in the AD brain (Fig. 1d, e). These retinal tau tangles were found in ganglion cells and βIII-tubulin^+^ cells within the INL of MCI and AD patients. Retinal tangles, as revealed by MC-1 and Bielschowsky silver staining, also appear as paperclip tau formations resembling NFTs (Fig. 1b,and e, right panel), as observed in the AD brain by Gallyas and Bielschowsky silver staining (Fig. 1d, ande, left panel; see additional Bielschowsky silver stain images across retinal layers and in paired brain tissues from other AD patients in Suppl. Fig. 1). Histological analysis of brain NFTs measured by Gallyas Silver and Thioflavin staining shows a significant 3.2-fold higher level in MCI and a 4.1-fold higher level in AD, compared to NC controls (Fig. 1f). Quantification of percent MC-1^+^ immunoreactive area in the respective retinas indicated significant and modest 2.0-fold and 1.8-fold increases in MCI and AD patients compared to NC controls, respectively (Fig. 1g). There was considerable overlap in the levels of retinal MC-1 percent area in aged individuals with normal cognition and those with MCI due to AD and AD dementia. Spearman’s rank correlation coefficient analysis demonstrated that retinal MC-1^+^ burden weakly associates with the severity of brain atrophy (Fig. 1h), as well as with NFTs, NTs, and cerebral amyloid angiopathy (CAA, Table 3).

### Identification of retinal tau oligomers with increases in MCI and AD patients linked to brain pathology, Braak staging, and cognitive status

**Fig. 2:**
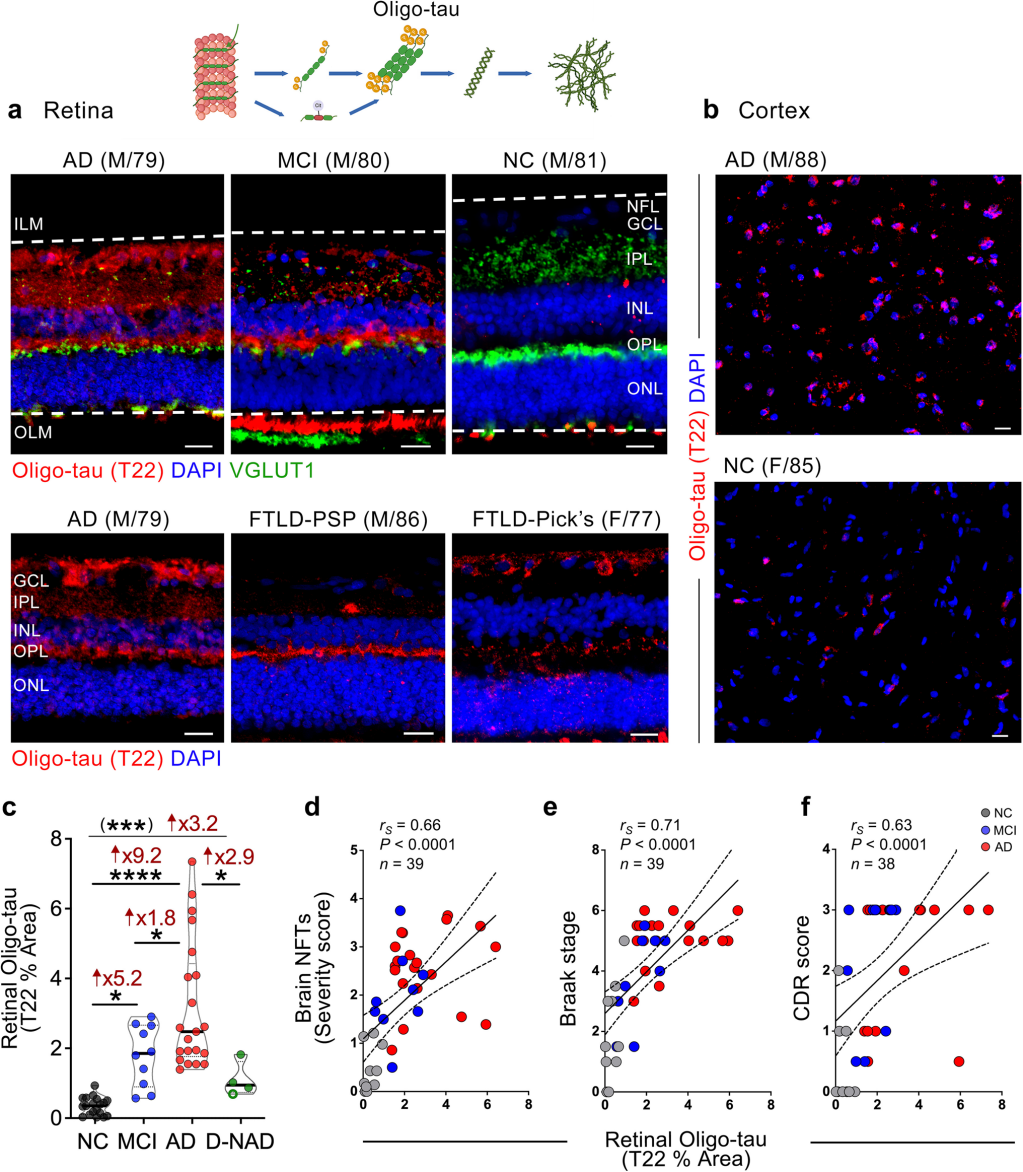
Identification of oligomeric tau in the retina of MCI, AD, and non-AD dementia patients. **a** Representative images of immunofluorescent staining for T22^+^ oligomeric tau (Oligo-tau, red), vesicular glutamate transporter 1 (VGLUT1, green), and nuclei (DAPI, blue) in retinal cross-sections from NC, MCI, and AD; representative images from patients with non-AD dementia (D-NAD), with frontotemporal lobar dementia (FTLD) with either progressive supranuclear palsy (PSP) or Pick’s disease, are also shown. Dashed lines demarcate the area of the quantitative IHC analysis, between the inner limiting membrane (ILM) and the outer limiting membrane (OLM). **b** Representative images of T22^+^ Oligo-tau immunofluorescence (red) and DAPI (blue) in cortical (A9) sections from NC and AD patients. Scale bars 20 µm. **c** Quantitative IHC analysis of the percent retinal T22^+^ Oligo-tau immunoreactive area (*n* = 19 NC, *n* = 10 MCI, *n* = 21 AD, and *n* = 4 D-NAD patients). Spearman's rank correlation coefficient analyses of retinal T22^+^ Oligo-tau against **d** brain NFTs severity score, **e** Braak stages, and **f** CDR scores. Individual data points (circles) and median, lower and upper quartile are shown in violin plots. *P < 0.05, ***P < 0.001, ****P < 0.0001, by one-way ANOVA and Tukey’s post-hoc multiple comparison test, or unpaired 2-tailed Student’s *t* test (in parenthesis). Fold changes are shown in red. D-NAD, non-AD dementia; F, female; M, male; age (in years); *n*, sample size. Illustrations created with Biorender.com

We next explored Oligo-tau forms in the retina of MCI and AD patients. In AD brains, toxic Oligo-tau forms are assembled from small p-tau aggregates after dislodging from microtubules in neurons and are shown to propagate from affected to unaffected brain regions [49, 64, 72]. Previous research demonstrated that extracting Oligo-tau from the AD brain using the T22 Ab and injecting these oligomers into wild-type mouse brains caused neurotoxicity and the propagation of abnormal endogenous murine tau [48]. Here, we performed anti-T22 immunolabeling in retinal and brain sections from a donor cohort comprising MCI (*n* = 10, mean age 88.7 ± 5.7 years, 6 females/4 males), AD (*n* = 21, mean age 86.1 ± 8.4 years, 11 females/10 males), and NC controls (*n* = 19, mean age 84.5 ± 9.1 years, 9 females/10 males). Additionally, we analyzed retinas from four cases of non-AD dementia (D-NAD; Fig. 2, extended data in Suppl. Fig. 2a). Compared with NC control retinas, we identified intense and diffuse-like T22^+^ Oligo-tau signals in the AD and MCI retinas, when labeled in combination with the pre-synaptic marker, vesicular glutamate transporter 1 (VGLUT1) and DAPI (blue) for nuclei (Fig. 2a, upper panel), or with DAPI (Fig. 2a, lower panel). In the retinas of MCI and AD patients compared to NC controls, we observed abundant cellular and diffused Oligo-tau, especially in the synaptic-rich layers (OPL, IPL), alongside scarce VGLUT1^+^ signals (Fig. 2a, upper panel). In comparison to retinas from AD dementia patients, retinal T22^+^ signals appeared fewer in D-NAD patients (Fig. 2a, lower panel, and Suppl. Fig. 2a). In the brain, the differences in T22^+^ Oligo-tau burden between AD and NC are also evident (Fig. 2b). Quantitative IHC analysis revealed a highly significant 9.2-fold increase in retinal T22^+^ Oligo-tau in AD patients and a significant 5.2-fold increase in MCI patients compared to NC controls. Retinal T22^+^ Oligo-tau burden in AD dementia patients was significantly 1.8 times higher compared to MCI patients and 2.9 times higher compared to D-NAD patients (Fig. 2c). There was a trend of elevated retinal Oligo-tau burden in D-NAD patients versus NC controls, which reached statistical significance by two-group analysis Student *t-test*. Notably, Spearman’s correlation analysis indicated that retinal T22^+^ Oligo-tau strongly and positively correlates with brain NFTs burden (Fig. 2d, *r*_*S*_ = 0.66, *P* < 0.0001), Braak staging—a parameter of tauopathy spread across brain regions during AD progression (Fig. 2e, *r*_*S*_ = 0.71, *P* < 0.0001), CAA severity (*r*_*S*_ = 0.69, *P* < 0.0001), and the A(amyloid-beta plaque) B(NFT stage) C(Neuritic plaque)—ABC scores (Table 3). Retinal Oligo-tau moderately correlates with brain Aβ burden and NTs. Furthermore, moderate to strong correlations were found with the MMSE and the CDR cognitive scores (Fig. 2f, Table 3; *r*_*S*_ = − 0.57, *P* < 0.001 and *r*_*S*_ = 0.63, *P* < 0.0001, respectively).

### GeoMx profiling of total tau and p-tau isoforms in the retina and brain of MCI and AD patients.

**Fig. 3:**
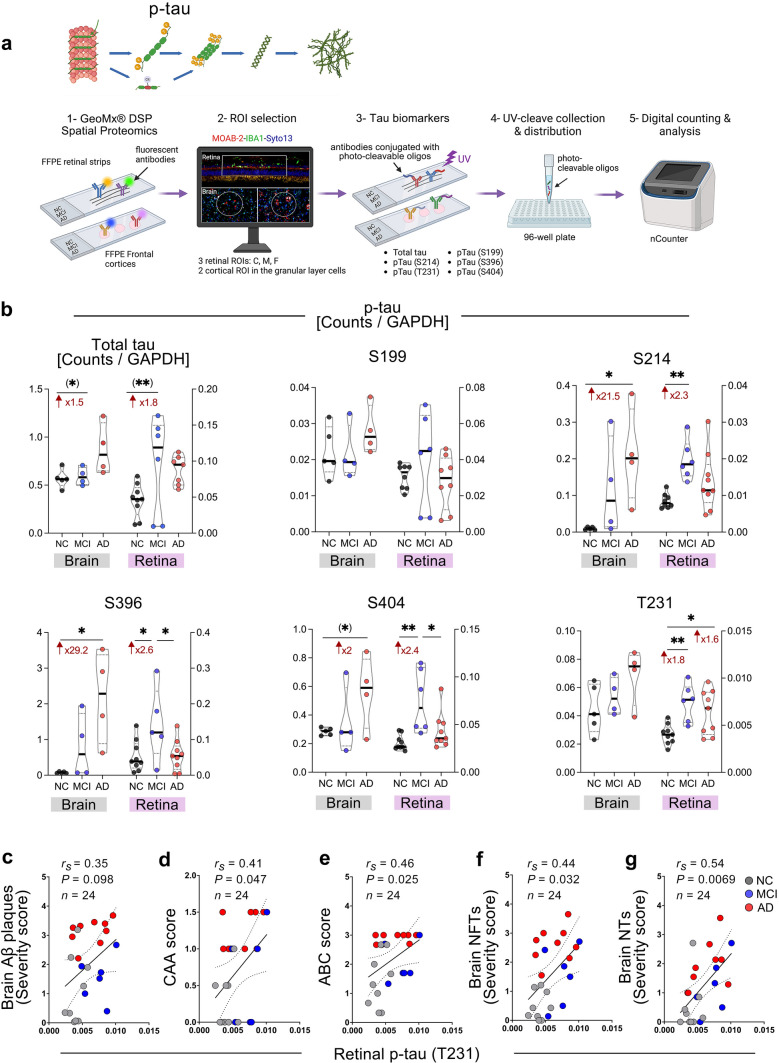
Tau isoforms quantified by GeoMx^®^ digital spatial profiling in retinas and brains from MCI and AD patients. **a** Graphical illustration of NanoString GeoMx^®^ digital spatial profiling (DSP) analyses for tau protein and tau isoforms in retinal and respective brain samples. **b** Quantitative analysis of retinal total tau and p-tau at phosphorylation sites of S199, S214, S396, S404, and T231 detected by GeoMx^®^ in retinas from AD (*n* = 9) and MCI (*n* = 6) patients, and NC controls (*n* = 9), and paired-brain tissues (frontal cortex region A9; *n* = 13 in total). Spearman’s rank correlation coefficient analyses are shown between retinal pT231-tau and the severity of brain **c** Aβ plaques, **d** CAA, **e** ABC, **f** NFTs, and **g** NTs scores. Individual data points (circles) and median, lower and upper quartile are shown in violin plots; *n*, sample size. *P < 0.05, **P < 0.01, by one-way ANOVA and Sidak’s post-hoc multiple comparison test for group analyses. Two group comparisons by unpaired 2-tailed Student *t* test are indicated in parenthesis. Fold changes are shown in red. Illustrations created with Biorender.com

We employed the high-throughput NanoString GeoMx^®^ digital spatial profiling (DSP) technique (Fig. 3a) to determine quantities of total tau protein and various p-tau forms in retinal cross-sections and corresponding brain cortical sections prepared from a donor cohort comprising MCI (retina: *n* = 6, mean age 88.5 ± 5.0 years, 3 females/3 males; brain: *n* = 4, mean age 87.5 ± 5.8 years, 3 females/1 male), AD (retina: *n* = 9, mean age 85.1 ± 7.8 years, 5 females/4 males; brain: *n* = 4, mean age 86.75 ± 4.3 years, 3 females/1 male), and NC controls (retina: *n* = 9, mean age 89.3 ± 9.4 years, 6 females/3 males; brain: *n* = 5, mean age 90.4 ± 7.3 years, 3 females/2 males). The list of individual patients is detailed in Supplementary Table 1.

The GeoMx tau module included the analysis of total tau and p-tau isoforms at sites of serine 199 (S199), serine 214 (S214), serine 396 (S396), serine 404 (S404), and threonine 231 (T231) (Fig. 3b). In the AD retinas and corresponding brains, there were trends of higher total tau levels compared to NC controls, which reached statistical significance for the AD brain by Student *t* test. While no difference was detected in total tau levels in the MCI brains versus NC controls, the total tau levels in the MCI retinas were 1.8-fold higher, reaching statistical significance by Student *t* test (Fig. 3b; top left). Significant increases in brain pS214- (21.5-fold), pS396- (29.2-fold), and pS404- (twofold) tau forms were found in AD patients compared to NC controls, while brain p-tau forms at sites S199 and T231 showed a non-significant trend of increases in the AD patients. For the MCI brain, only pS214- and pS396-tau forms showed a non-significant trend of increases compared to NC controls, and the other forms showed no differences.

In the retina, significant increases in retinal S214 (2.3-fold), S396 (2.6-fold), S404 (2.4-fold), and T231 (1.8-fold) p-tau forms were detected in MCI patients compared to NC controls. Retinal pT231-tau was significantly elevated (1.6-fold) in AD patients compared to NC controls (Fig. 3b). Interestingly, the levels of retinal pS396- and pS404-tau were significantly higher in MCI versus AD patients. Furthermore, our quantitative GeoMx analysis in this cohort indicated that retinal pT231-tau significantly and weakly correlated with brain Aβ plaques and moderately correlated with CAA, ABC, brain NFTs, and brain NTs severity scores (Fig. 3c–g).

### Histological evaluation of total tau and p-tau isoforms in the retina of MCI and AD patients

**Fig. 4:**
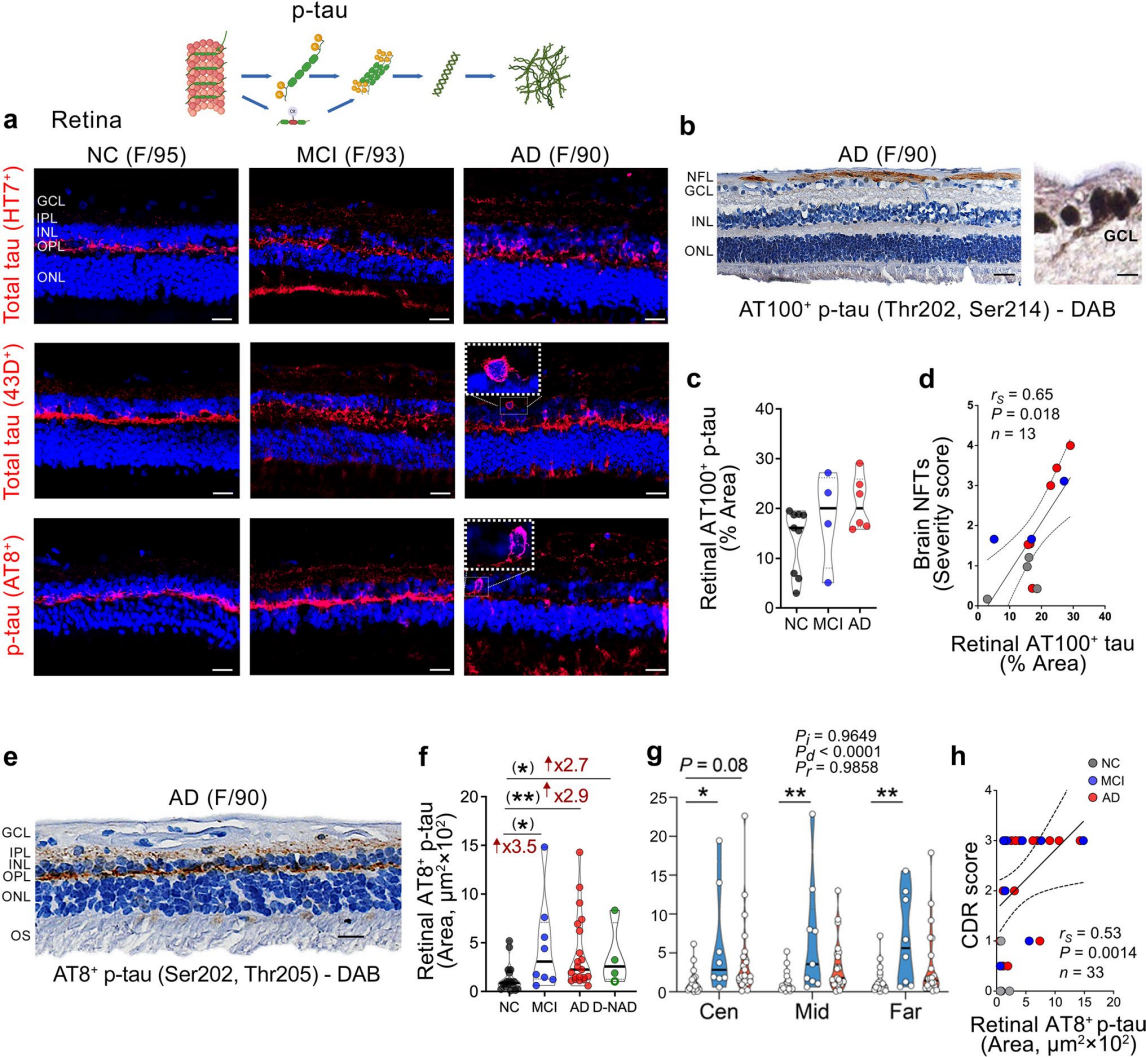
Total tau, AT100-positive, and AT8-positive p-tau isoforms detected by histological examination of retinas from MCI and AD patients. **a** Representative images of immunofluorescent staining for total tau (HT7^+^ and 43D^+^) and AT8^+^ p-tau (S202/T205) in retinal cross-sections from human donors with NC, MCI, and AD. Scale bars 20 µm. Additional inserts of high-magnification images show pronounced intra-neuronal 43D^+^ tau and AT8^+^ p-tau staining in the AD retina. **b** Representative images of peroxidase-based DAB staining of retinal AT100^+^ p-tau (T202/S214) in an AD patient; a high magnification image for the ganglion cell layer (GCL). **c** Quantitative analysis of percent retinal AT100^+^ p-tau immunoreactive area (*n* = 9 NC, *n* = 4 MCI, and *n* = 6 AD). **d** Spearman's rank correlation coefficient analyses between retinal AT100^+^ p-tau and brain NFTs severity scores. **e** Representative images of peroxidase-based DAB staining of AT8^+^ p-tau (S202/T205) in retinal cross-sections from AD patients. **f** Quantitative analysis of retinal AT8^+^ p-tau immunoreactive area (*n* = 18 NC, *n* = 8 MCI, *n* = 21 AD, and *n* = 4 D-NAD). **g** Mapping of AT8^+^ p-tau in central (Cen), Mid-, and Far-peripheral retinal subregions in the same cohort. **h** Spearman's rank correlation coefficient analysis between retinal AT8^+^ p-tau and the cognitive status as assessed by CDR scores. Individual data points (circles) and median, lower and upper quartile are shown in violin plots. *P < 0.05, **P < 0.01, by one-way ANOVA and Tukey’s or Sidak’s multiple comparison tests, or unpaired 2-tailed Student *t* test for two group analysis (in parenthesis). Fold changes are shown in red. F, female; M, male; age (in years); *n*, sample size. Illustrations created with Biorender.com

**Fig. 5:**
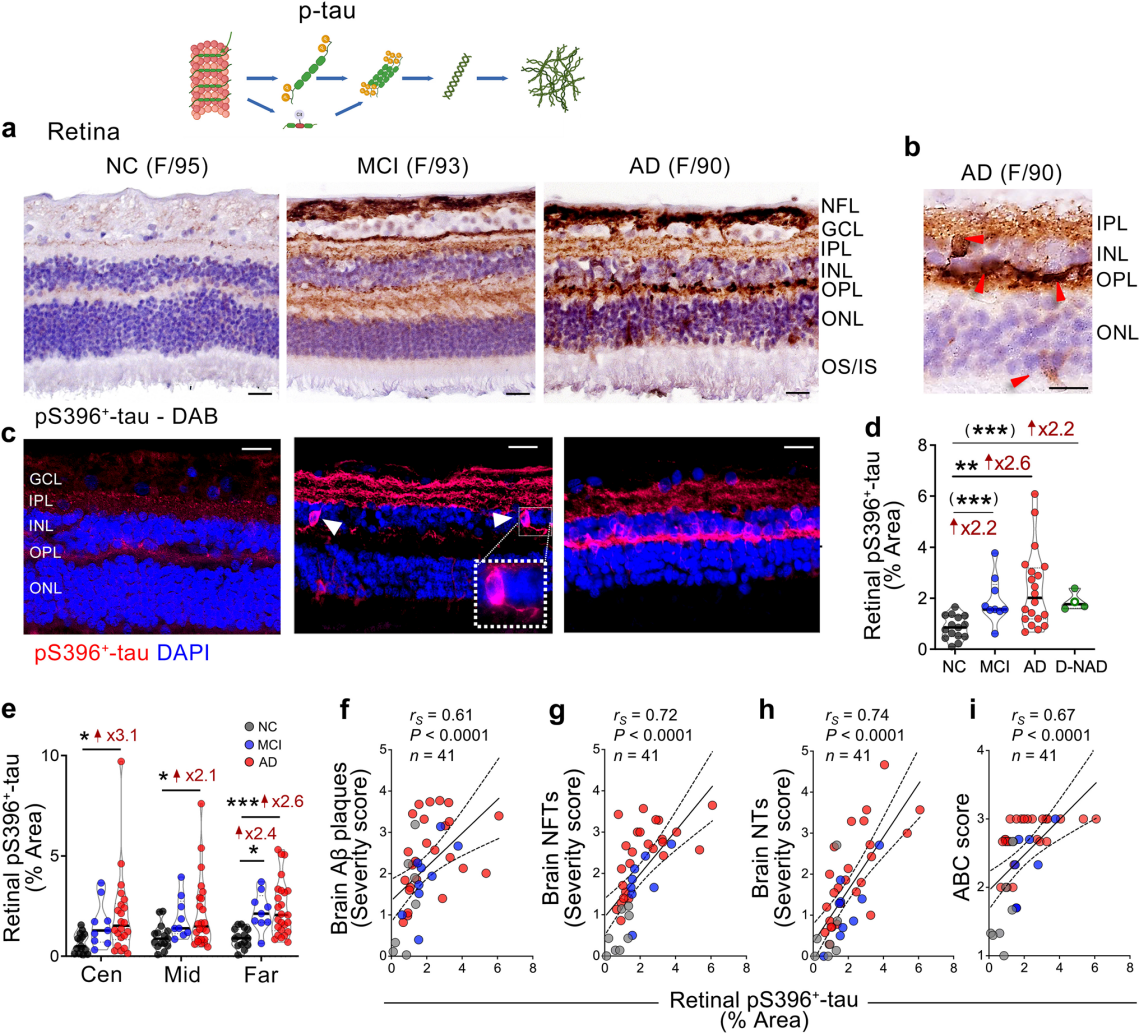
Retinal pS396-tau in MCI and AD patients. **a–c** Representative images of immunofluorescent and peroxidase-based staining of pS396-tau in retinal cross-sections from AD and MCI patients as compared to NC controls. Scale bars 20 µm. Higher magnification images show intracellular pS396-tau in the INL and ONL (**b** red arrowheads and **c** white arrowheads). **d** Quantitative analysis of the percent pS396-tau immunoreactive area in the retina (*n* = 15 NC, *n* = 9 MCI, *n* = 25 AD, and *n* = 4 D-NAD). **e** Percent pS396-tau area separated to central (Cen), Mid-, and Far-peripheral retinal subregions in the same cohort. Spearman's rank correlation coefficient analyses are shown between retinal pS396-tau and the severity of brain **f** Aβ plaques, **g** NFTs, **h** NTs, and **i** ABC scores. Individual data points (circles) and median, lower and upper quartile are shown in violin plots. *P < 0.05, **P < 0.01, ***P < 0.001, by one-way ANOVA and Tukey’s post-hoc multiple comparison test, or unpaired 2-tailed Student *t* test for two group analysis (in parenthesis). Fold changes are shown in red. F, female; M, male; age (in years); *n*, sample size. Illustrations created with Biorender.com

The detection of significant changes in retinal and brain tau isoforms in GeoMx DSP analysis prompted an additional histological examination of total tau and other retinal p-tau forms at epitopes T202/S214 (AT100^+^), S202/T205 (AT8^+^), and S396 (pS396^+^), in larger cohorts (Figs. 4 and 5; extended data in Suppl. 2–4). IHC analysis of total tau using both HT7 and 43D antibodies revealed a considerable retinal tau signal, mostly restricted to the OPL in NC subjects, and across all retinal layers in MCI and AD patients (Fig. 4a, upper and middle panels). Analysis of retinal AT100^+^ p-tau forms was performed in a subset cohort consisting of AD (*n* = 6, mean age 80.67 ± 14.73 years, all females), MCI (*n* = 4, mean age 92.75 ± 4.99 years, all females), and NC controls (*n* = 9, mean age 81.22 ± 12.20 years, 8 females/1 male). Our histological analysis showed retinal AT100-positive immunoreactivity in the innermost retinal layers, including nerve fiber layer (NFL) and GCL (Fig. 4b), and a non-significant trend of increases in MCI and AD patients compared to NC controls (Fig. 4c). Retinal AT100^+^ p-tau forms were strongly associated with the NFTs burden (Fig. 4d, Table 3).

We next examined retinal AT8^+^ p-tau signals (Fig. 4a, lower panel and Fig. 4e) in a subset of donors with MCI (*n* = 8, mean age 89.75 ± 5.50 years, 4 females/4 males), AD (*n* = 21, mean age 82.81 ± 13.40 years, 10 females/11 males), and NC controls (*n* = 18, mean age 81.50 ± 8.96 years, 9 females/9 males). AT8^+^ pS202/T205-tau isoforms were frequently detected in the OPL, and to a lesser extent, in the IPL (Fig. 4a, e); a staining pattern that is comparable with previous reports in the AD retina [34, 87]. Quantitative IHC analysis revealed a 3.5-fold trend of increases in MCI and a 2.9-fold in AD, compared to NC controls (Fig. 4f), reaching significance by Student’s *t* test. Retinal AT8^+^ p-tau burden in D-NAD patients was at similar levels as those observed in MCI and AD patients and had a trend of a 2.7-fold increase compared to NC controls (Fig. 4f; representative images in Suppl. Fig. 2b). Examination of AT8^+^ signals in the central, mid-, and far-peripheral retina indicated significant increases in MCI compared to NC in all three retinal subregions (Fig. 4g). Retinal AT8^+^ p-tau forms significantly and weakly associated with brain NFTs, ABC, and CAA severity scores, while showing a moderate correlation with the CDR cognitive scores (Fig. 4h, Table 3).

Analysis of retinal pS396^+^ tau was performed in a cohort consisting of donors with MCI (*n* = 9, mean age 89.67 ± 5.15 years, 5 females/4 males), AD (*n* = 25, mean age 86.80 ± 8.25 years, 16 females/9 males), and NC controls (*n* = 15, mean age 84.33 ± 8.96 years, 6 females/9 males). Using immunofluorescence and peroxidase-based immunostaining, we found substantial pS396-tau depositions across all retinal layers in MCI and AD patients compared to NC controls (Fig. 5a–c; arrowheads indicate intraneuronal p-tau structures). In agreement with the quantitative GeoMx DSP findings (Fig. 3b), stereological analysis of pS396-tau revealed a 2.2-fold increase in MCI compared to NC retinas, reaching significance by Student *t* test. The AD retinas exhibited a significant 2.6-fold higher pS396-tau burden compared to NC controls (Fig. 5d). Retinas from D-NAD patients have on average, similar levels of pS396-tau burden as those observed in MCI and AD patients (Fig. 5d; representative images in Suppl. Fig. 3a), with a trend of 2.2-fold increase compared to NC retinas, reaching significance by Student *t* test. Examination of retinal pS396-tau isoforms per retinal subregion indicates that the far-peripheral retina exhibits an earlier and more significant increase of these p-tau isoforms, providing clearer separation between the diagnostic groups (Fig. 5e; representative images per retinal central, mid- and far-peripheral subregions in Suppl. Fig. 4). Notably, strong and highly significant Spearman’s correlations were identified between retinal pS396-tau burden and brain Aβ plaques, and moreover, NFTs and NTs severity scores (Fig. 5f–h, *P* < 0.0001; *r*_*S*_ = 0.61, *r*_*S*_ = 0.72, and *r*_*S*_ = 0.74, respectively). Moderate-to-strong correlations were also found between retinal pS396-tau and ABC scores (Fig. 5i) and Braak staging, with a weak correlation to the CDR cognitive scores (Table 3).

### Retinal PHF-tau increases in AD dementia patients and correlates with brain tauopathy

**Fig. 6:**
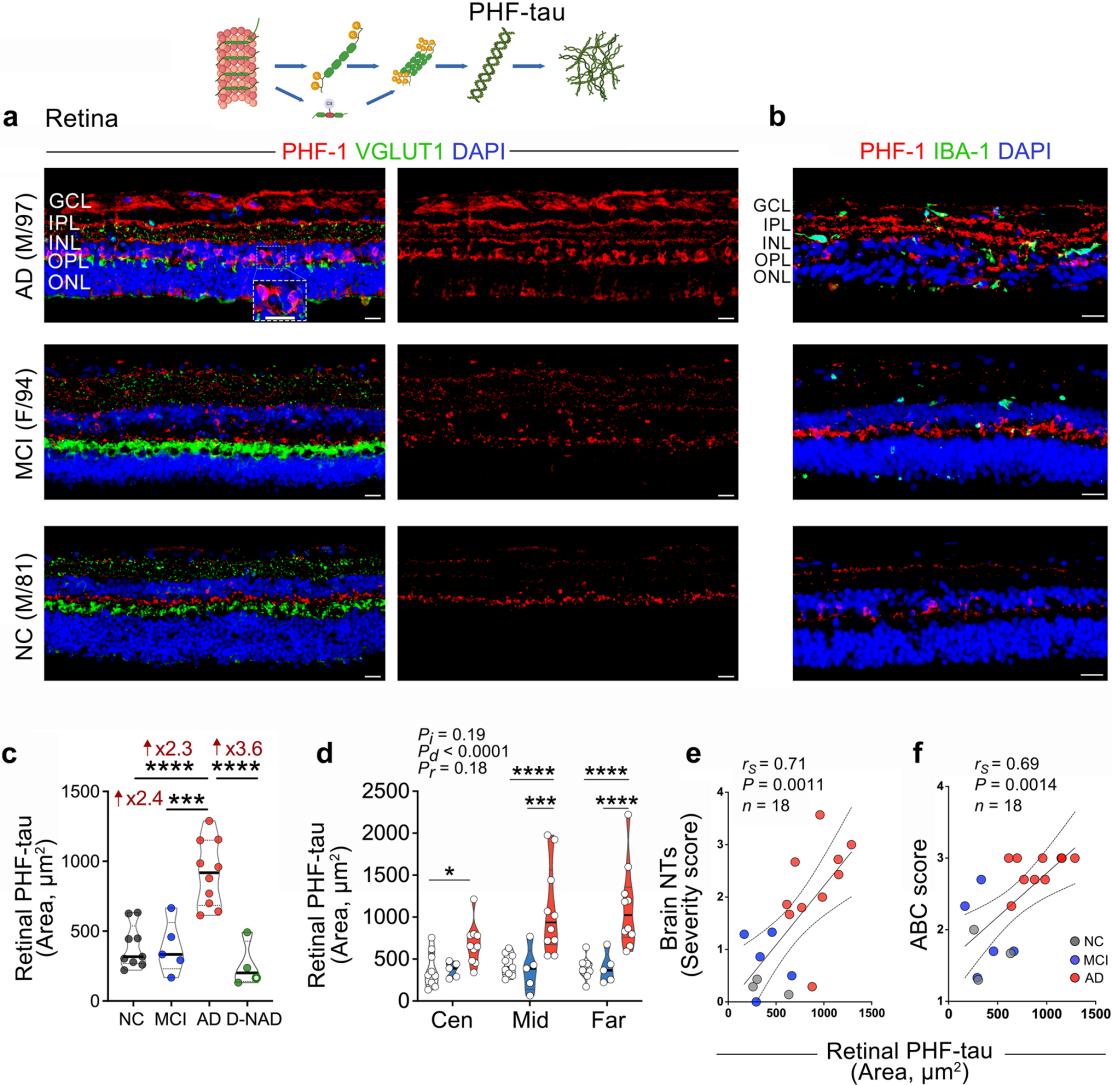
Paired-helical filaments of tau in retinas of MCI and AD patients. **a**, **b** Representative images of immunofluorescent stainings for PHF-1^+^ paired-helical filaments of tau (PHF-tau, red), vesicular glutamate transporter 1 (VGLUT1) or IBA1 (green), and DAPI (blue) in retinal cross-sections from patients with MCI and AD, and NC controls. Scale bars 20 µm. **c** Quantitative analysis of retinal PHF-tau immunoreactive area (*n* = 9 NC, *n* = 5 MCI, *n* = 10 AD, and *n* = 4 D-NAD). **d** Mapping of PHF-tau area in central (Cen), Mid-, and Far-peripheral retinal subregions in the same cohort. Spearman's rank correlation coefficient analyses are shown between retinal PHF-tau and the severity of brain **e** NTs and **f** ABC scores. Individual data points (circles) and median, lower and upper quartile are shown in violin plots. *P < 0.05, ***P < 0.001, ****P < 0.0001, by one-way ANOVA and Tukey’s or Sidak’s multiple comparison post-tests for group analyses. Fold changes are shown in red. F, female; M, male; age (in years); *n*, sample size. Illustrations created with Biorender.com

Upon detecting increased levels of p-tau, Oligo-tau, and tau tangle forms in the retina of MCI and AD patients, we further examined the pre-NFT forms—the PHF-tau aggregates. Previously, brain PHF-tau in AD has been associated with chronic neuroinflammation, including activated microgliosis [53, 85]. Additionally, tau-laden neurons were susceptible to excessive microglial synaptic pruning [85]. Retinal IHC analysis, using the PHF-1 antibody recognizing pS396- and pS404-tau in paired helical filaments, was performed on a subset of patient donors with MCI (*n* = 5, mean age 89.8 ± 5.8 years, 3 females/2 males), AD (*n* = 10, mean age 88.1 ± 7.4 years, 5 females/5 males), and NC controls (*n* = 9, mean age 82.2 ± 7.9 years, 3 females/6 males) (Fig. 6). This analysis showed marked PHF-tau deposition across retinal layers in AD patients, particularly localized in VGLUT1^+^ synaptic-rich OPL and IPL, alongside INL and GCL, and IBA1^+^ microgliosis-laden regions (Fig. 6a, b). Quantitative IHC analysis revealed a highly significant 2.3-fold increase in retinal PHF-tau in AD, but not in MCI, compared to NC controls (Fig. 6c). Notably, retinal PHF-tau burden in AD dementia patients was significantly 2.4 times elevated compared to MCI patients and 3.6 times higher compared to D-NAD patients (Fig. 6c; representative images for donors with D-NAD in Suppl. Fig. 3b), with no overlap between the AD and D-NAD groups. Examination of retinal PHF-tau distribution per retinal subregion indicates that the mid- and far-peripheral retina show more significant increases of PHF-tau forms in AD patients versus MCI and NC controls (Fig. 6d). Retinal PHF-tau deposition strongly associated with brain NTs burden and ABC scores (Fig. 6e, f; *r*_*S*_ = 0.71, *P* = 0.0011 and *r*_*S*_ = 0.69, *P* = 0.0014, respectively), and moderately with Braak staging, CAA, brain atrophy, and NFTs severity scores (Table 3).

### Identification of citrullinated-tau forms in the MCI and AD retinas and association to cognition

**Fig. 7:**
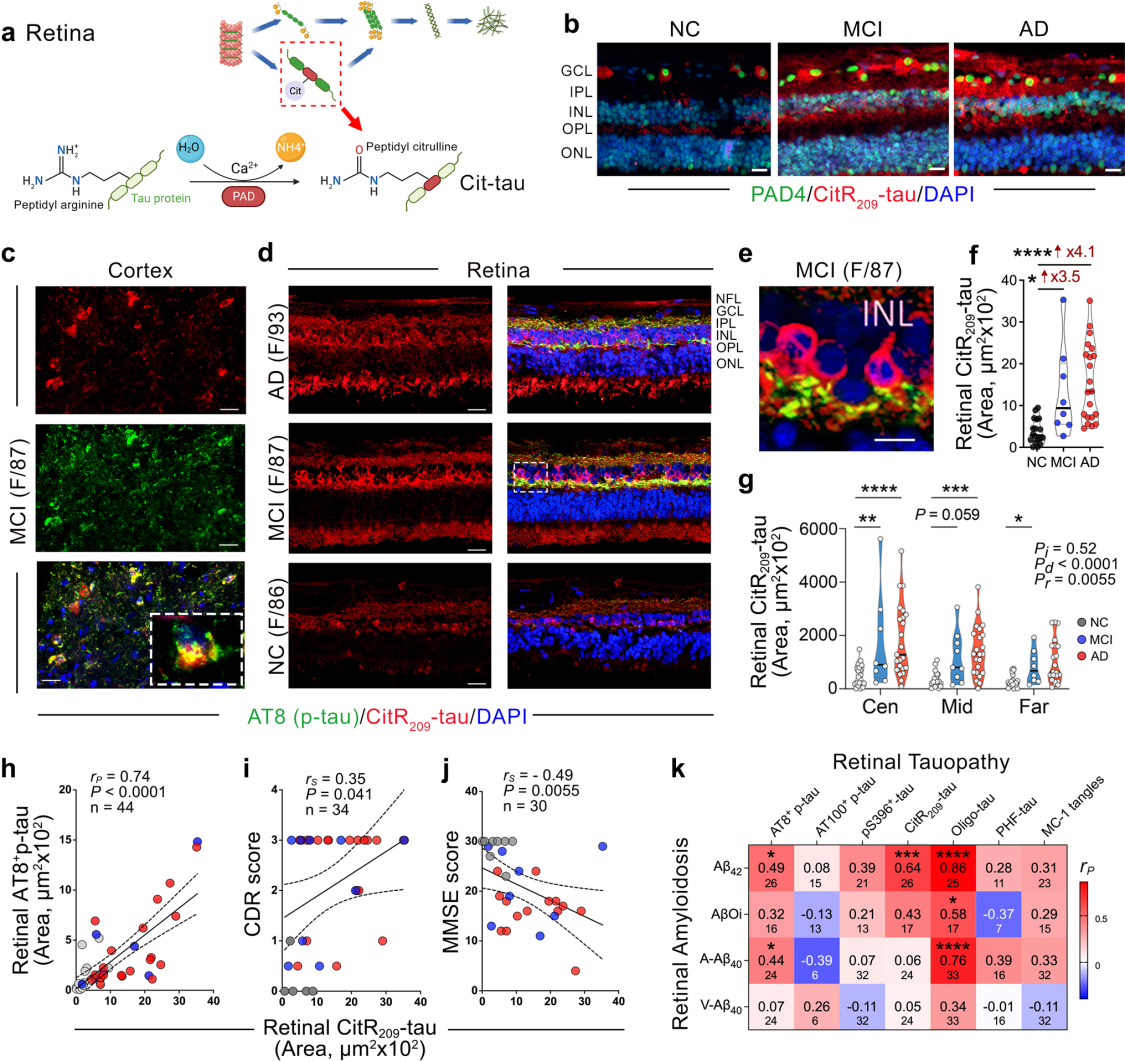
Identification of citrullinated-tau isoforms in the MCI and AD retinas. **a** Graphical illustration of post-translation modification of tau citrullination, catalyzed by protein arginine deiminase (PAD) enzymes. **b** Representative images of immunofluorescent staining for PAD4 enzyme (green), citrullinated tau at arginine R209 site (CitR_209_-tau, red), and nuclei (DAPI, blue) in retinal cross-sections from MCI and AD patients as compared with NC controls. Scale bars 20 µm. **c-e** Representative images of immunofluorescent staining for AT8^+^p-tau (green), CitR_209_-tau (red), and DAPI (blue) in **c** brain cortex (A9) and **d**,** e** retinal cross-sections from patients with AD, MCI, and NC controls. **e** High-magnification image shows intra-cellular CitR209-tau labeling in retinal INL of MCI patient. Scale bars 20 µm. **f** Quantitative analysis of retinal CitR_209_-tau immunoreactive area (*n* = 18 NC, *n* = 8 MCI, and *n* = 21 AD). **g** Mapping of CitR_209_-tau area in central (Cen), Mid-, and Far-peripheral retinal subregions in the same cohort. **h** Pearson’s correlation coefficient (*r*) between retinal CitR_209_-tau and retinal AT8^+^ p-tau. Spearman's rank correlation coefficient analyses are shown between retinal CitR_209_-tau and **i** CDR and **j** MMSE cognitive scores. **k** Heatmap of Pearson’s correlation coefficient (*r*) analyses between retinal Aβ forms [12F4^+^-Aβ_42_, intraneuronal scFvA13^+^-Aβ oligomers (AβOi), arterial (A) 11A50-B10^+^-Aβ_40_, and venular (V) 11A50-B10^+^-Aβ_40_] and retinal tau isoforms [AT8^+^, AT100^+^, and pS396-p-tau, CitR_209_-tau, Oligo-tau, PHF-tau, and MC-1 tau tangles]. The strength (darker color) and direction (positive-red, negative-blue) of the correlations are shown. Pearson’s *r*_*P*_ values are indicated in larger fonts and below are the number (*n*) of pair-wise correlations. Individual data points (circles) and median, lower and upper quartile are shown in violin plots. *P < 0.05, **P < 0.01, ***P < 0.001, ****P < 0.0001, by one-way ANOVA and Tukey’s or Sidak’s multiple comparison post-tests for group analyses. Fold changes are shown in red. F, female; M, male; age (in years); *n*, sample size. Illustrations created with Biorender.com

Citrullination is a post-translational modification in which an arginine amino acid is converted to a citrulline amino acid (Fig. 7a). This process is catalyzed by peptidyl arginine deiminase (PAD) enzymes, which play a significant role in several chronic diseases [18]. Neurons expressing PAD4 were found to accumulate citrullinated proteins in AD cortices and hippocampi [2]. A recent study identified altered PAD4 expression and Cit-tau accumulation in the brains of AD patients [52]. Aberrant tau deposition activated PAD4 in neurons, leading to the citrullination of tau at multiple arginine residues. Here, we identified prominent neuronal PAD4 expression along with marked depositions of retinal and cortical citrullinated arginine (R)-209 tau (CitR_209_-tau) and AT8^+^p-tau (S202/T205) in MCI and AD patients compared to NC controls (Fig. 7b–d). In both retinal and paired brain tissues, we found increased co-localized signals of AT8^+^ p-tau and CitR_209_-tau in MCI and AD patients, with clear intra-neuronal CitR_209_-tau signals in the INL (Fig. 7e; higher magnification of MCI retina). We conducted quantitative analysis of retinal CitR209-tau in a cohort of donors with MCI (*n* = 8, mean age 89.13 ± 5.22 years, 4 females/4 males), AD (*n* = 21, mean age 82.81 ± 13.40 years, 10 females/11 males), and NC controls (*n* = 18, mean age 80.72 ± 7.89 years, 9 females/9 males). Stereological quantification of retinal CitR_209_-tau indicates a substantial 3.5-fold and 4.1-fold increases in MCI and AD patients, respectively, compared to NC controls (Fig. 7f). Analysis of CitR_209_-tau burden per retinal subregion indicated that CitR_209_-tau appears earlier and more pronouncedly in the central retina (Fig. 7g). Moreover, Pearson’s correlation analysis indicated a strong positive association between the two post-translational modifications of tau (CitR_209_-tau and AT8^+^p-tau) in the retina (Fig. 7h; *r*_*P*_ = 0.74, *P* < 0.0001). Spearman’s rank correlation analyses demonstrated weak-to-moderate associations between retinal CitR_209_-tau and CDR or MMSE cognitive scores (Fig. 7i, j), with no associations to the severity of brain pathology or disease staging (Table 3).

We then investigated the relationships between the various retinal tau isoforms and retinal Aβ alloforms; the latter recently investigated by our group [44, 73]. To achieve this, we performed pair-wise Pearson’s correlation analyses between retinal tau isoforms (AT8^+^, AT100^+^, and pS396-p-tau, CitR_209_-tau, Oligo-tau, PHF-tau, and MC-1 tau tangles) and retinal Aβ species (12F4^+^-Aβ_42_, intraneuronal scFvA13^+^-Aβ oligomers (AβOi), arterial (A) 11A50-B10^+^-Aβ_40_, and venular (V) 11A50-B10^+^-Aβ_40_) (Fig. 7k). A very strong and significant correlation was found between retinal Oligo-tau and retinal Aβ_42_ forms (*r*_*P*_ = 0.86, *P* < 0.0001) and retinal arterial Aβ_40_ burden (*r*_*P*_ = 0.76, *P* < 0.0001). Retinal CitR_209_-tau strongly correlated with retinal Aβ_42_ burden (*r*_*P*_ = 0.64, *P* < 0.001). Moderate linear associations were found between Oligo-tau and retinal AβOi as well as between retinal AT8^+^-p-tau and retinal Aβ_42_ and arterial Aβ_40_ alloforms (Fig. 7k).

## Discussion

In this study, we present the first evidence of retinal oligomeric and citrullinated tau isoforms, alongside increases in MC-1^+^ tau tangles, primarily pretangles, in the retina of MCI and AD patients. We found that pretangle forms of p-tau (at epitopes S202/T205, S214, S396, S404, T231, but not S199 and S212/T214), CitR_209_-tau, and T22^+^ Oligo-tau, as well as MC-1^+^ pretangle and mature tau tangles, are elevated in the retinas of patients with the earliest functional impairment (MCI due to AD). Notably, retinal PHF-tau increases only in the later stages of AD dementia. Interestingly, retinas from non-AD dementia patients (DLB, FTLD) exhibited lower Oligo-tau and PHF-tau burdens than AD dementia patients, suggesting these retinal tau isoforms as potential markers of differential diagnosis for dementia types. GeoMx spatial profiling analyses reveal increases in total tau and various site-specific tau phosphorylation epitopes in the AD retina, particularly at the MCI stage. Importantly, retinal tau oligomers exhibited the largest magnitude of increases in MCI and further in AD patients compared to matched NC controls, showing the strongest correlations with Braak staging, which represents the spread of brain tauopathy, as well as with CAA and CDR cognitive function. Retinal pS396-tau demonstrated the strongest associations with brain NTs and NFTs severity scores. Retinal MC-1-positive stainings detected scarce paperclip tau formations, resembling brain NFTs [57], in MCI and AD patients. Only pS396-tau and Oligo-tau forms in the retina correlated with brain Aβ plaque burden, and retinal mature tau tangles, recognized by PHF-1 and MC-1 antibodies, correlated with brain atrophy. Our analysis also suggested a close interaction between tau oligomers and Aβ_42_ and arterial Aβ_40_ species in the AD retina, in agreement with studies in the AD brain showing the interconnection between Aβ and tau accumulation, along with Aβ-induced acceleration of tau spreading via neuronal communications [19, 84]. Overall, our data demonstrate that most tauopathy isoforms are increased in the AD and MCI (due to AD) retina and correlate with one or more AD neuropathology and cognitive parameters.

Aggregates of p-tau, in the form of brain NFTs, are core hallmarks that define AD diagnosis and are tightly linked to neurodegeneration [38, 39, 90]. Consistent with this, our study detected PHF-tau and MC-1^+^ mature tau tangles in the retinas of MCI and AD patients. These tau isoforms were the only abnormal forms significantly correlated with the severity of brain atrophy. Despite some contradictions, several previous studies have reported an association between neocortical NFT burden and antemortem cognitive decline in AD cases [10, 29, 54, 61, 62, 81]. In our cohort, there were modest but significant increases in retinal MC-1-positive tau forms in MCI and AD patients, with no association with cognitive status, as assessed by CDR or MMSE scores. This suggests that these pretangle and mature tau tangle forms may not affect the retina to the same degree as the brain during AD progression.

Previous studies have described the accumulation of diverse p-tau forms and NFT-like structures in postmortem retinal tissues from AD patients [20, 21, 31, 34, 43, 65, 70, 87]. A recent study reported a mild MC-1-positive signal in retinal OPL, INL, and IPL from AD cases, which specifically recognizes conformational paperclip tau folding [87]. However, none have successfully detected the typical formation of mature fibrillary tau forms of PHF-tau or NFTs in the retina. In our study, immunohistochemical staining using the PHF-1 and MC-1 antibodies, and the Bielschowsky silver stain, validated the presence of typical paired-helical filaments of tau, and scarcely, the paperclip structures resembling NFTs in the retina of MCI and AD patients. These tau forms are primarily found within neurons in the INL and GCL. Inconsistencies in previous observations likely stem from variations in the geometric regions that were analyzed, as well as tissue preservation, fixation, and staining protocols [3, 27].

Oligomers of tau are considered highly neurotoxic intermediate assemblies that are precursors of protofilaments, PHF-tau, and subsequent NFTs [72]. In the AD brain, tau oligomers spread across anatomical regions and are involved in the early stages of AD pathogenesis [48, 72]. These oligomers have been shown to impair neuronal energy production, synaptic integrity, microtubule assembly, and axonal transport [64]. In this study, we identified a substantial accumulation of T22-positive tau oligomers in the retina of MCI and AD patients compared to normal controls. Both diffused and intracellular tau oligomer signals were observed across the retinal layers, predominantly spanning from the OPL through the innermost retinal layers. Among the diverse retinal tau isoforms measured in this study, tau oligomers exhibited the most extensive increases in MCI and AD retinas, showing strong associations with brain NFTs, Braak staging, and CDR cognitive scores. Therefore, tau oligomers should be evaluated as a potential retinal tau marker for detection and tracking of AD progression in future studies. Notably, the unexpected strong associations with CAA severity and retinal arterial Aβ_40_ deposits merit further investigation of the possible interactions between tau oligomer buildup and the accumulation of arterial amyloidosis.

Previous studies utilizing IHC and western blot analyses on postmortem retinas from AD donors have identified retinal total tau (HT7 clone) and p-tau forms at multiple phosphorylation sites, including S202, T205, T217, T212, S214, T181, T231, S396, and S404 [20, 21, 31, 34, 65, 70, 87]. In this study, we employed the GeoMx spatial profiling method and detected increased total tau in the AD brains and MCI retinas, aligning with previous findings in the brain [86]. Quantitative GeoMx analysis also indicated elevated levels of retinal p-tau at S214, S396, S404, and T231 sites in MCI patients compared to controls, with moderate associations between retinal pT231-tau and brain ABC, CAA, NFTs, and NTs severity scores. This is consistent with a previous study showing increased pT231-tau levels in the CSF of preclinical AD patients [78]. Interestingly, retinal pS396- and pS404-tau levels were significantly higher in MCI patients compared to AD dementia patients, suggesting their earlier accumulation during AD progression. Histological examinations revealed strong correlations between additional retinal p-tau types and brain pathology or cognitive status. Retinal AT8-positive pS202/T205-tau burden moderately correlated with CDR cognitive scores, whereas AT100-positive pS212/T214-tau, and particularly, pS396-tau forms, strongly associated with brain NFTs burden. Indeed, retinal pS396-tau exhibited highly significant and strong associations with the core AD pathologies and disease staging. Of note, a previous study reported that retinal AT8^+^ pS202/T205-tau correlated with brain NFTs levels in the hippocampus, temporal pole, medial frontal gyrus, and parietal lobe [34]. Confirming our findings, previous studies have shown the sensitivity to detect the pretangle forms, pS202/T205-tau (with AT8 clone) and pSer396-tau (with several clones), in retinas from AD patients [20, 21, 34, 65, 70, 87]. Therefore, retinal pS202/T205-tau, pT231-tau, and pS396-tau forms should be further considered as potential markers to track brain NFTs severity and cognitive decline. Collectively, these results suggest that retinal p-tau accumulation may occur early in AD pathogenesis and may serve as predictors of brain tauopathy and cognitive status.

In a previous study, retinal PHF-tau isoforms were reported in the IPL, OPL, and INL of AD patients [87]. We observed that PHF-tau is not restricted to these three layers but also extends to the retinal NFL, GCL, and ONL in AD patients. The abnormal folding of p-tau at both S396 and S404 sites leads to the generation of insoluble PHF-tau [30]. While GeoMx data show upregulation of pS396- and pS404-tau isoforms in MCI retinas, histological analysis of conformational PHF-tau structures indicated increases later in the AD dementia retina compared to control retinas. This result indicates that, unlike the early hyperphosphorylation of tau, its folding into PHF-tau may occur in the retina at later disease stages. Correlation analyses further suggest that retinal PHF-tau may be a moderate to strong predictor for tracking brain NFTs, NTs, and ABC scores in patients.

The current study provides the first evidence of hyper-citrullinated tau in the human MCI and AD retina. Post-translational modifications of tau are prerequisites for the formation of PHF-tau and NFTs [28]. Tau is a substrate of the PAD4 enzyme, which can cause irreversible citrullination of its arginine residues [52]. In our study, we identified increases in retinal CitR_209_-tau in MCI, and further, in AD patients. In the OPL and INL, we observed a considerable amount of co-localization and close interplay between retinal CitR_209_-tau and AT8-positive pS202/T205-tau species, suggesting that citrullination and hyperphosphorylation both occur during the development of retinal tauopathy in AD. Furthermore, similar to retinal AT8-positive pS202/T205-tau, retinal CitR_209_-tau exhibited significant correlations with the cognitive status. Since hyper-citrullination of proteins has been implicated in multiple chronic human diseases [5, 18] and citrullinated tau may impact oligomerization and microglial activation [52], our findings of increased retinal CitR_209_-tau may imply altered accumulation or clearance properties of tau in the MCI and AD retina.

This study provides insights into various aberrant tau forms associated with AD in the retinas of both MCI and AD patients and defines their relationship to disease status. However, we acknowledge a few limitations. This is a cross-sectional case–control study primarily focused on correlations; therefore, caution must be exercised before implicating cause-and-effect conclusions. Furthermore, the lack of clinical information on visual system-related symptoms hinders our ability to assess potential connections between retinal tauopathy and visual dysfunction. This underscores the need for future studies to explore the relationships between retinal tauopathy, retinal neurodegeneration, and ocular outcomes in patients. In addition, this study identifies tauopathy in retinal tissues from MCI (due to AD) patients and non-AD dementia patients with DLB and FTLD with tauopathy versus subjects with AD dementia and normal cognition. Nevertheless, we recognize that a larger sample size in ethnically diverse populations is warranted in future studies.

A recent study proposes the spread of AT8^+^ p-tau from the OPL to the IPL/GCL in the retina of AD patients [87]. In agreement, our representative images from NC, MCI, and AD patients may suggest the spread of retinal tau isoforms from OPL to IPL/GCL (AT8^+^ p-tau, T22^+^ Oligo-tau, PHF-tau) or from NFL/GCL/IPL to OPL (pS396-tau). Furthermore, most tau forms seem to spread from peripheral to central ST/IT retinal subregions, while CitR_209_-tau appears to spread from central to peripheral ST/IT retina. However, this is speculative for the current study and merits future quantitative investigation of the spatiotemporal distribution of tau isoforms in different retinal quadrants and layers during disease stages to confirm these observations. In addition, given the inconsistencies in previous reports on retinal AD tauopathy—where several studies identified certain pathological tau forms in the AD retina [21, 31, 34, 65, 87] that two earlier reports could not detect [36, 88] or could partially detect [20, 70]—workshop groups focused on harmonizing the methodologies for analyzing retinal tauopathy will be useful in future research [27].

In summary, our study provides evidence of diverse and novel pathological tau isoforms in the retinas of MCI and AD patients. These include classical conformational tau aggregates such as PHF, pretangles and mature paperclip tau tangles, Oligo-tau, p-tau, and Cit-tau forms. Our findings indicate that the retina is affected by tauopathy during AD pathogenesis and that aberrant retinal tau forms may serve as predictors of brain tauopathy, disease staging, and cognitive decline. Collectively, this study offers valuable insights into AD-related retinal tau forms, which have the potential to be utilized in the future development of noninvasive, high spatial-resolution retinal tau imaging tools for the early detection and monitoring of AD.

### Supplementary Information

Below is the link to the electronic supplementary material.

Supplementary file1 (PDF 10213 KB)

## Abbreviations

A: Asian
Ab: Antibody
ABC: Amyloid/Braak/CERAD score
AD: Alzheimer’s disease
ADRC: Alzheimer’s Disease Research Center
Aβ: Amyloid β-protein
Aβ_40_: 40 Amino acid-long alloform
Aβ_42_: 42 Amino acid-long alloform
AβOi: Intraneuronal Aβ oligomers
ALS: Amyotrophic lateral sclerosis
ANOVA: Analysis of variance
APP: Amyloid precursor protein
AT8: Anti-tau antibody recognizing phosphorylation at serine 202 and threonine 205
AT100: Anti-tau antibody recognizing phosphorylation at serine 214 and threonine 212
B: Black
C (Cen): Central retina
CA: Cornu ammonis
CAA: Cerebral amyloid angiopathy
CDR: Clinical Dementia Rating
Cit-tau: Citrullinated tau
CitR_209_-tau: Citrullinated arginine (R)-209 tau
CNS: Central nervous system
43D: Anti-tau antibody
DAB: Diaminobenzidine
DLB: Dementia with Lewy bodies
D-NAD: Non-AD dementia
DSP: Digital spatial profiler
F: Far peripheral retina
FTLD: Frontotemporal lobar dementia
GCL: Ganglion cell layer
H: Hispanic
HT7: Anti-tau antibody
IBA1: Ionized calcium binding adaptor molecule 1
IHC: Immunohistochemistry
ILM: Inner limiting membrane
INL: Inner nuclear layer
IPL: Inner plexiform layer
IR area: Immunoreactive area
IT: Inferior-temporal
M: Mid peripheral retina
mAb: Monoclonal antibody
MC-1: Monoclonal antibody that identifies the amino acid (aa) 7‐9 and 312‐322 tau epitopes
MCI: Mild cognitive impairment
MMSE: Mini Mental State Examination
NDRI: National Disease Research Interchange
NC: Normal cognition
NFL: Nerve fiber layer
NFT: Neurofibrillary tangle
NT: Neuropil thread
OD: Optic disc
Oligo-tau: Oligomeric tau
OLM: Outer limiting membrane
ONL: Outer nuclear layer
OPL: Outer plexiform layer
OR: Outer retina
PAD: Peptidyl arginine deiminase
PAD4: Peptidyl arginine deiminase 4
PHF: Paired helical filament
PMI: Postmortem interval
PSP: Progressive supranuclear palsy
p-tau: Hyperphosphorylated tau
ROI: Region of interest
SD: Standard deviation
S199: Serine 199
S214: Serine 214
pS396: Anti-tau antibody recognizing phosphorylation at serine 296 epitope
S404: Serine 404
ST: Superior-temporal
T22: Anti-tau oligomers antibody
T231: Threonine 231
ThioS: Thioflavin-S
VGLUT1: Vesicular glutamate transporter
W: White
