# Supplementary material for: Identification of retinal oligomeric, citrullinated, and other tau isoforms in early and advanced AD and relations to disease status
Source: Acta Neuropathol. 2024 Jul 9;148(1):3. doi: 10.1007/s00401-024-02760-8 (PMC11233395; doi:10.1007/s00401-024-02760-8)
Supplement: Supplementary file 1 — Supplementary file1 (PDF 10213 KB) [file 401_2024_2760_MOESM1_ESM.pdf]

**Supplementary Online Content**

**Identification of retinal oligomeric, citrullinated, and other tau isoforms in early and advanced AD and relations to disease status**.

Haoshen Shi, Nazanin Mirzaei, Yosef Koronyo, Miyah R. Davis, Edward Robinson, Gila M. Braun, Ousman Jallow, Altan Rentsendorj, V Krishnan Ramanujan, Justyna Fert-Bober, Andrei A. Kramerov, Alexander V. Ljubimov, Lon S. Schneider, Warren G. Tourtellotte, Debra Hawes, Julie A. Schneider, Keith L. Black, Rakez Kayed, Maj-Linda B. Selenica, Daniel C. Lee, Dieu-Trang Fuchs, and Maya Koronyo-Hamaoui

Corresponding author: Maya Koronyo-Hamaoui, PhD, Cedars-Sinai Medical Center, 127 S. San Vicente Blvd., A6212, Los Angeles, CA, USA 90048. Tel: (310)-423-7473, E-mail: maya.koronyo@csmc.edu

**Supplementary Table 1.** List of human donors in this study.

**Supplementary Table 2.** List of antibodies.

**Supplementary Figure 1.** Bielschowsky silver staining for brain NFTs and retinal mature tau tangles.

**Supplementary Figure 2.** Immunofluorescent staining for Oligo-tau and AT8$^+$ p-tau in non-AD dementia patients.

**Supplementary Figure 3.** Immunofluorescent staining for pS396$^+$-tau and PHF-tau in non-AD dementia patients.

**Supplementary Figure 4.** Regional differences in retinal pS396-tau.

**Supplementary Table 1. List of human donors in this study.**

| Diagnosis | Sex | Race | Age at death | Thal A | Braak B | CERAD C | CAA Score | Co-morbid. [LB/ASVD] | Braak Stage | CDR Score | MMSE Score | APOE status | Study type |
|---|---|---|---|---|---|---|---|---|---|---|---|---|---|
| AD1 | F | W | 93 | 2 | 3 | 3 | 1 | −/+ | V | 3 | n.a. | n.a. | IHC |
| AD2 | M | H | 97 | 3 | 2 | 3 | 1 | −/+ | III | 1 | 26 | e3/e3 | IHC |
| AD3 | M | W | 79 | 3 | 3 | 3 | 1.5 | −/+ | V | n.a. | n.a. | n.a. | IHC |
| AD4 | F | W | 87 | 3 | 3 | 3 | n.a. | −/+ | V-VI | 3 | n.a. | e3/e4 | IHC |
| AD5 | M | W | 88 | 2 | 3 | 2 | 1 | −/+ | V-VI | 1 | 18 | e3/e4 | IHC |
| AD6 | M | W | 77 | 3 | 3 | 3 | 1 | −/− | VI | 2 | 18 | e3/e4 | IHC |
| AD7 | F | W | 66 | 3 | 3 | 3 | 0 | −/+ | VI | 3 | 2 | e3/e3 | IHC |
| AD8 | F | H | 99 | 3 | 2 | 3 | 1.5 | −/− | IV | 3 | 23 | e3/e3 | IHC |
| AD9* | F | H | 81 | 3 | 3 | 3 | 1.5 | −/+ | V-VI | 3 | 12 | e3/e3 | IHC/GeoMx |
| AD10* | F | W | 90 | 3 | 3 | 3 | 1 | −/+ | V-VI | 3 | n.a. | e3/e4 | IHC/GeoMx |
| AD11* | M | W | 90 | 3 | 2 | 3 | 1 | −/+ | III-IV | 3 | n.a | e3/e4 | IHC/GeoMx |
| AD12 | M | W | 90 | 3 | 3 | 3 | 1 | −/+ | VI | 3 | n.a. | n.a. | IHC/GeoMx |
| AD13 | F | W | 90 | 1 | 3 | 3 | 1 | −/+ | V | 2 | 9 | n.a. | IHC/GeoMx |
| AD14 | M | W | 79 | 2 | 2 | 2 | 2 | −/+ | V | 0.5 | 24 | n.a. | IHC |
| AD15 | F | H | 92 | 2 | 2 | 2 | 2 | −/+ | III | 3 | 9 | e3/e3 | IHC |
| AD16 | M | W | 88 | 3 | 3 | 3 | 1.5 | −/+ | V-VI | 1 | 16 | e2/e3 | IHC/GeoMx |
| AD17 | F | B | 94 | 3 | 3 | 3 | 0 | −/+ | V-VI | 3 | n.a. | e3/e3 | IHC |
| AD18 | F | W | 87 | 2 | 3 | 3 | 1.5 | −/+ | V | 3 | 16 | e3/e4 | IHC |
| AD19 | F | W | 70 | 3 | 3 | 3 | 1.5 | −/+ | V | 0.5 | 24 | n.a. | IHC |
| AD20 | M | A | 81 | 3 | 3 | 3 | 1 | −/− | V | 3 | 12 | e4/e4 | IHC |
| AD21 | M | W | 92 | 3 | 3 | 3 | 0 | −/+ | VI | 3 | 15 | e3/e3 | IHC |
| AD22 | M | W | 66 | 3 | 3 | 3 | 1.5 | −/n.a. | V | 3 | 19 | n.a. | IHC/GeoMx |
| AD23 | M | A | 40 | 3 | 2 | 3 | 1 | −/− | III-IV | 3 | n.a. | e3/e3 | IHC |
| AD24 | F | A | 88 | 2 | 3 | 3 | 1.5 | −/+ | V | 3 | 4 | n.a. | IHC |
| AD25 | F | W | 100 | 2 | 3 | 3 | 1 | −/+ | VI | 2 | 16 | n.a. | IHC |
| AD26* | F | W | 86 | 3 | 3 | 2 | 1 | −/+ | V-VI | 3 | 18 | e3/e4 | IHC/GeoMx |
| AD27 | F | W | 85 | 3 | 3 | 3 | 1.5 | −/+ | V-VI | 3 | n.a. | e3/e3 | IHC/GeoMx |
| AD28 | F | A | 93 | 2 | 2 | 2 | 1.5 | +/+ | III-IV | 3 | 17 | n.a. | IHC |
| AD29 | F | W | 76 | 3 | 3 | 3 | 2 | −/+ | V | 1 | 26 | e3/e4 | IHC |
| AD30 | F | A | 93 | 3 | 2 | 2 | 0 | −/+ | III-IV | 3 | 20 | e3/e3 | IHC |
| AD31 | F | W | 97 | 3 | 3 | 3 | 0 | −/+ | IV | n.a. | 4 | n.a. | IHC |
| AD32 | F | W | 63 | 3 | 3 | 3 | 0 | −/+ | V | 2 | 16 | n.a. | IHC |
| AD33 | F | W | 65 | 3 | 3 | 3 | 2 | −/+ | V-VI | 3 | 2 | n.a. | IHC |
| AD34 | M | W | 90 | 3 | 3 | 2 | 2 | −/+ | V | n.a. | n.a. | n.a. | IHC |

| Diagnosis | Sex | Race | Age at death | Thal A | Braak B | CERAD C | CAA Score | Co-morbid. [LB/ASVD] | Braak Stage | CDR Score | MMSE Score | APOE status | Study type |
|---|---|---|---|---|---|---|---|---|---|---|---|---|---|
| MCI1 | M | W | 97 | 2 | 3 | 3 | 1 | −/+ | V | 1 | 28 | e3/e3 | IHC |
| MCI2* | M | H | 80 | 3 | 3 | 2 | 1 | −/+ | V | 3 | 29 | e3/e3 | IHC/GeoMx |
| MCI3* | F | B | 94 | 2 | 1 | 2 | 0 | n.a./− | I-II | 0.5 | 29 | e3/e3 | IHC/GeoMx |
| MCI4* | F | W | 89 | 1 | 2 | 2 | 1 | −/+ | III-IV | 0.5 | 24 | e3/e3 | IHC/GeoMx |
| MCI5 | F | W | 93 | 3 | 2 | 2 | 2 | −/+ | IV | 3 | 11 | e3/e3 | IHC |
| MCI6 | M | W | 93 | 2 | 0 | 2 | 0 | −/+ | 0 | 3 | 19 | e2/e3 | IHC/GeoMx |
| MCI7 | F | W | 86 | 3 | 1 | 3 | 0 | −/+ | I-II | 2 | 15 | e3/e4 | IHC |
| MCI8 | M | W | 88 | 1 | 2 | 2 | 0 | −/+ | III | 3 | n.a. | n.a. | IHC/GeoMx |
| MCI9 | F | W | 80 | 3 | 3 | 3 | 1 | +/n.a. | V | 3 | n.a. | n.a. | IHC |
| MCI10* | F | W | 87 | 3 | 3 | 3 | 1.5 | −/+ | V-VI | 3 | 13 | e3/e3 | IHC/GeoMx |
| MCI11 | F | W | 98 | 2 | 3 | 2 | 1.5 | −/+ | V | 2 | 15 | n.a. | IHC |
| NC1 | F | W | 93 | n.a. | n.a. | n.a. | n.a. | n.a. | n.a. | n.a. | n.a. | n.a. | IHC |
| NC2 | F | W | 86 | n.a. | n.a. | n.a. | n.a. | n.a. | n.a. | n.a. | n.a. | n.a. | IHC |
| NC3 | M | W | 78 | n.a. | n.a. | n.a. | n.a. | n.a. | n.a. | n.a. | n.a. | n.a. | IHC |
| NC4 | M | H | 76 | 2 | 0 | 2 | 0 | −/+ | 0 | 0 | 29 | e3/e3 | IHC |
| NC5* | M | W | 95 | 1 | 1 | 1 | 0 | −/− | I | 0 | 30 | e3/e3 | IHC/GeoMx |
| NC6 | F | W | 88 | n.a. | n.a. | n.a. | n.a. | n.a. | n.a. | n.a. | n.a. | n.a. | IHC |
| NC7* | M | H | 81 | 3 | 1 | 2 | 0 | −/+ | I-II | 0 | 23 | e3/e4 | IHC/GeoMx |
| NC8* | F | W | 99 | 1 | 2 | 1 | 0 | −/− | III | 0 | n.a. | e3/e3 | IHC/GeoMx |
| NC9* | F | W | 92 | 1 | 1 | 0 | 0.5 | −/+ | I | n.a. | 25 | n.a. | IHC/GeoMx |
| NC10 | M | W | 69 | 0 | 0 | 1 | 0 | −/+ | 0 | 1 | 28 | n.a. | IHC/GeoMx |
| NC11 | F | W | 91 | 2 | 2 | 2 | 0 | −/+ | III | 2 | 29 | n.a. | IHC |
| NC12 | M | W | 77 | n.a. | n.a. | n.a. | n.a. | n.a. | n.a. | n.a. | 30 | n.a. | IHC |
| NC13 | M | W | 73 | n.a. | n.a. | n.a. | n.a. | n.a. | n.a. | n.a. | n.a. | n.a. | IHC |
| NC14 | M | W | 84 | n.a. | n.a. | n.a. | n.a. | n.a. | n.a. | n.a. | 30 | n.a. | IHC |
| NC15 | M | W | 70 | n.a. | n.a. | n.a. | n.a. | n.a. | n.a. | n.a. | n.a. | n.a. | IHC |
| NC16 | F | W | 95 | 3 | 3 | 2 | 1 | −/+ | V | 0 | 30 | e3/e3 | IHC/GeoMx |
| NC17 | F | W | 93 | 3 | 2 | 3 | 0.5 | −/+ | III-IV | 1 | 27 | e2/e3 | IHC/GeoMx |
| NC18* | F | H | 85 | 2 | 1 | 2 | 0 | −/+ | I-II | 0 | 30 | e3/e3 | IHC/GeoMx |
| NC19 | M | B | 80 | n.a. | n.a. | n.a. | n.a. | n.a. | n.a. | n.a. | 29 | n.a. | IHC |
| NC20 | M | W | 58 | n.a. | n.a. | n.a. | n.a. | n.a. | n.a. | n.a. | 29 | n.a. | IHC |
| NC21 | M | W | 75 | n.a. | n.a. | n.a. | n.a. | n.a. | n.a. | n.a. | n.a. | n.a. | IHC |
| NC22 | M | W | 87 | n.a. | n.a. | n.a. | n.a. | n.a. | n.a. | n.a. | 30 | n.a. | IHC |

| Diagnosis | Sex | Race | Age at death | Thal A | Braak B | CERAD C | CAA Score | Co-morbid. [LB/ASVD] | Braak Stage | CDR Score | MMSE Score | APOE status | Study type |
|---|---|---|---|---|---|---|---|---|---|---|---|---|---|
| NC23 | M | W | 75 | n.a. | n.a. | n.a. | n.a. | n.a. | n.a. | n.a. | n.a. | n.a. | IHC |
| NC24 | F | W | 76 | n.a. | n.a. | n.a. | n.a. | n.a. | n.a. | n.a. | n.a. | n.a. | IHC |
| NC25 | F | W | 75 | n.a. | n.a. | n.a. | n.a. | n.a. | n.a. | n.a. | n.a. | n.a. | IHC |
| NC26 | F | W | 76 | n.a. | n.a. | n.a. | n.a. | n.a. | n.a. | n.a. | n.a. | n.a. | IHC |
| NC27 | F | W | 77 | n.a. | n.a. | n.a. | n.a. | n.a. | n.a. | n.a. | n.a. | n.a. | IHC |
| NC28 | F | W | 71 | n.a. | n.a. | n.a. | n.a. | n.a. | n.a. | n.a. | n.a. | n.a. | IHC |
| NC29 | F | B | 73 | n.a. | n.a. | n.a. | n.a. | n.a. | n.a. | n.a. | n.a. | n.a. | IHC |
| NC30 | F | W | 95 | 1 | 0 | 0 | 0.5 | −/− | I | 0 | 30 | n.a. | IHC/GeoMx |
| DLB - *sparce NFT* | M | W | 79 | 3 | 0-1 | 3 | 0 | +/− | 0-I | 3 | n.a. | e3/e3 | IHC |
| FTLD/ALS C90rf72 - *sparce NFT* | M | W | 66 | 0 | 0-1 | 0 | 0 | −/+ | 0-I | n.a. | n.a. | n.a. | IHC |
| FTLD-Pick's disease - *tauopathy, no NFT* | F | W | 77 | 0 | 0 | 0 | 0 | −/+ | 0 | n.a. | n.a. | n.a. | IHC |
| FTLD-PSP - *tauopathy, no NFT* | M | W | 86 | 0 | 0 | 0 | 0 | −/+ | 0 | n.a. | n.a. | n.a. | IHC |

AD, Alzheimer's disease dementia; MCI, mild cognitive impairment; NC, normal cognition; F, female; M, male; A, Asian; B, Black; H, Hispanic; W, White; IHC, Immunohistochemistry; A, Aβ plaque score modified from Thal; B, NFT stage modified from Braak; C, Neuritic plaque score modified from CERAD; CAA, Cerebral amyloid angiopathy; LB, Lewy bodies; ASVD. Atherosclerosis; CDR, Clinical dementia rating; MMSE, Mini-Mental State Examination; n.a., not available; +: present; –: none; APOE, apolipoprotein alleles; DLB, Dementia with Lewy bodies; FTLD, Frontotemporal lobar degeneration, a pathological process that occurs in frontotemporal dementia; ALS, Amyotrophic Lateral Sclerosis; PSP, progressive supranuclear palsy; *: donors with retina and paired corresponding brain used for GeoMx spatial profiling.

**Supplementary Table 2.** List of antibodies.

| Antibodies or Reagents | Source Species | Dilution | Application | Commercial Source | Catalog. # |
|---|---|---|---|---|---|
| *Primary antibody* | | | | | |
| MC-1 mAb | Mouse | 1:200 | IF, DAB | Peter Davis Lab | - |
| Anti-tau T22 pAb | Rabbit | 1:200 | IF | Rakez Kayed Lab | - |
| Tau Monoclonal Antibody (HT7) | Mouse | 1:500 | IF | ThermoFisher | MN1000 |
| Purified anti-Tau, 1-100 Antibody (43D) | Mouse | 1:40 | IF | Biolegend | 816601 |
| Phospho-tau (Thr212, Ser214) mAb (AT100) | Mouse | 1:100 | IF, DAB | ThermoFisher | MN1060 |
| Phospho-tau (Ser202, Thr205) mAb (AT8) | Mouse | 1:250 | IF, DAB | ThermoFisher | MN1020 |
| Phospho-tau (Ser396) pAb | Rabbit | 1:1500 | IF, DAB | Anaspec | AS-54977 |
| Tau phos Ser396/Ser404 mAb (PHF-1) | Mouse | 1:200 | IF | Peter Davis Lab | - |
| $Cit_{209}$tau | Mouse | 1:5000 | IF | Daniel Lee Lab | - |
| βIII-tubulin mAb | Mouse | 1:1000 | IF | Abcam | Ab78078 |
| PAD4 | Mouse | 1:100 | IF | Abcam/ Fert-Bober Lab | Ab128086 |
| Iba1 pAb | Rabbit | 1:200 | IF | FUJIFILM | 019-19741 |
| *Secondary antibody* | | | | | |
| Cy2 (anti-Rabbit) | Donkey | 1:200 | IF | Jackson ImmunoResearch Laboratories | |
| Cy3 (anti-rabbit, anti-goat, anti-mouse) | Donkey | 1:200 | IF | Jackson ImmunoResearch Laboratories | |
| Cy5 (anti-mouse) | Donkey | 1:200 | IF | Jackson ImmunoResearch Laboratories | |

Abbreviation: IF – immunofluorescence; DAB - peroxidase-based immunohistochemistry visualized with DAB substrate; pAb – polyclonal antibody; mAb – monoclonal antibody.

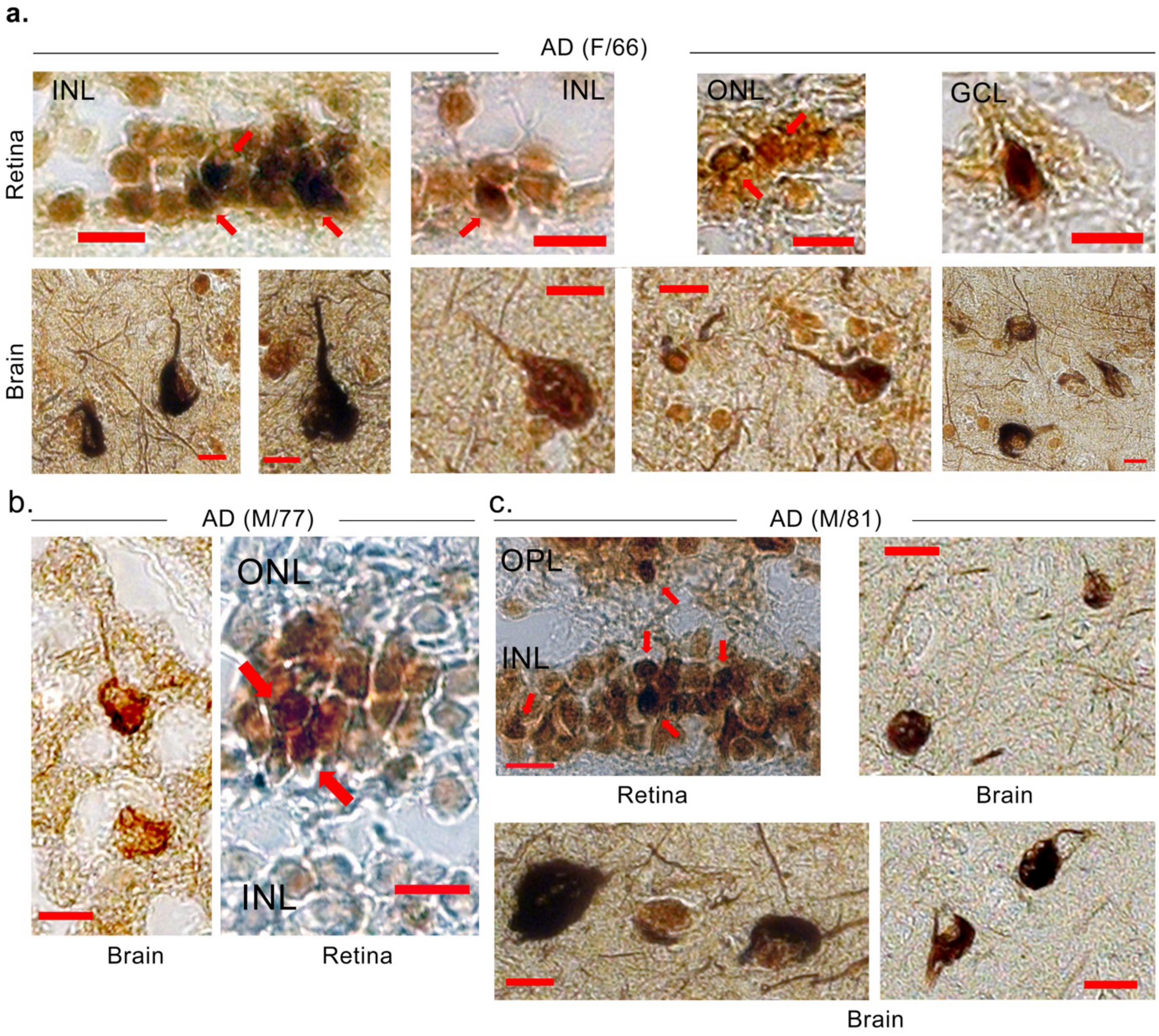


**Supplementary Figure 1.** Bielschowsky silver staining for brain NFTs and retinal mature tau tangles. **a-c**. Bielschowsky silver staining for brain NFTs and retinal mature tau tangles from 3 different AD patients. Scale bars=10µm.

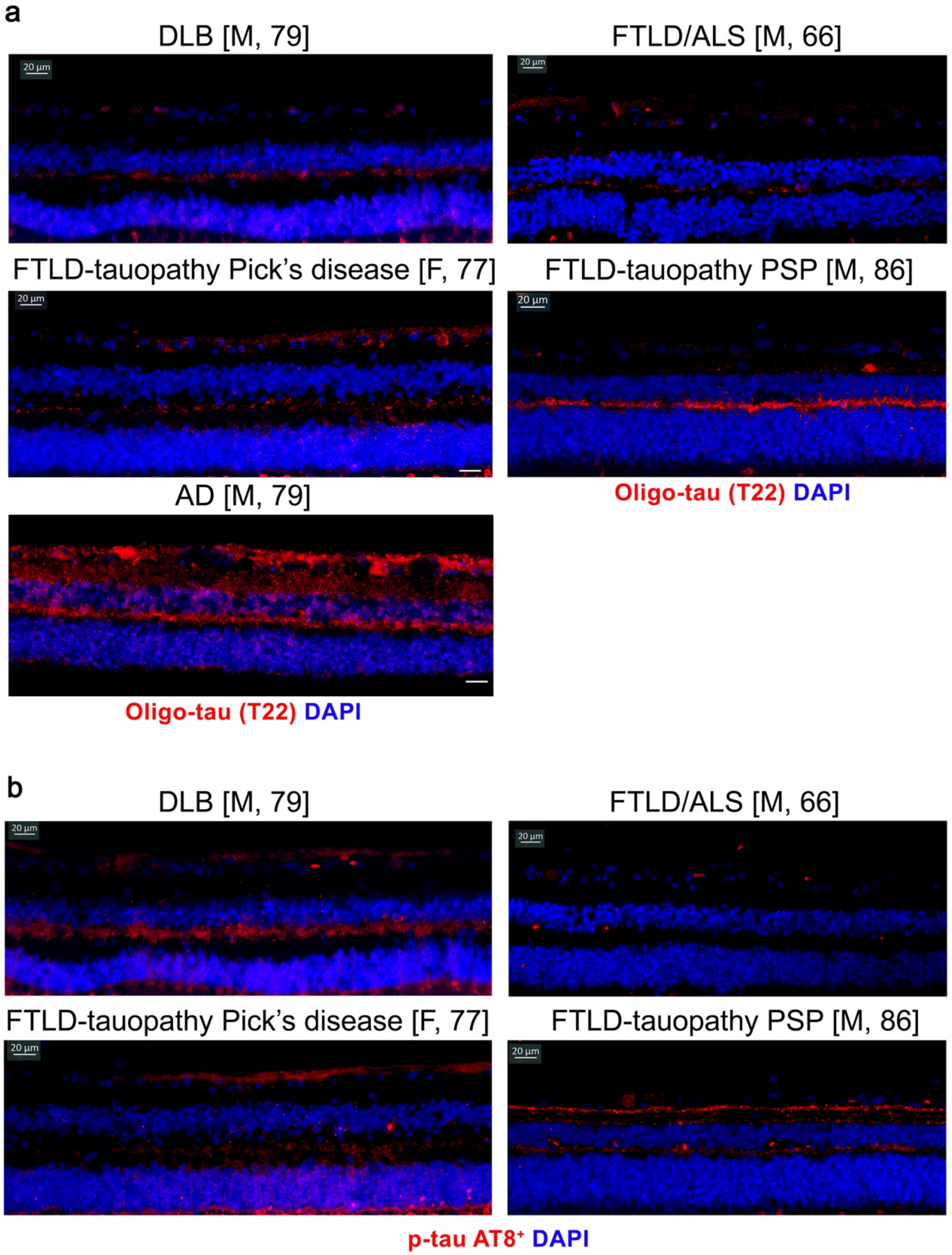


**Supplementary Figure 2.** Immunofluorescent staining for Oligo-tau and $AT8^+$ p-tau in non-AD dementia patients. Representative images of immunofluorescence staining of **a** $T22^+$-Oligo tau (red) and **b** $AT8^+$ p-tau (red) and nuclei (DAPI, blue) in patients with DLB, FTLD, and AD. AD, Alzheimer's disease dementia; DLB, Dementia with Lewy bodies; FTLD, Frontotemporal lobar degeneration with frontotemporal dementia; ALS, Amyotrophic Lateral Sclerosis; PSP, progressive supranuclear palsy. F, female; M, male; age (in years).

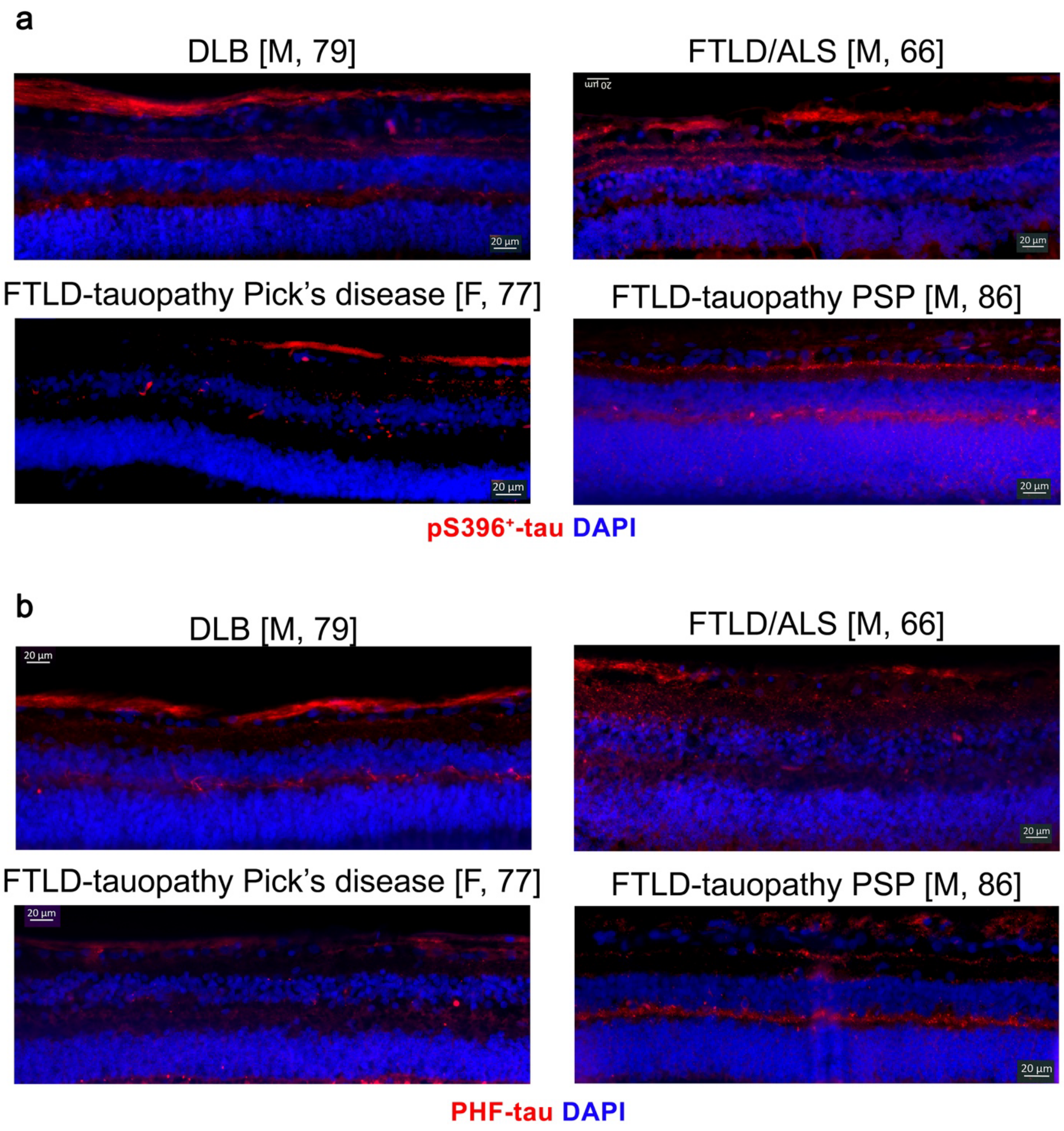


**Supplementary Figure 3.** Immunofluorescent staining for pS396$^+$-tau and PHF-tau in non-AD dementia patients. Representative images of immunofluorescence staining of **a** pS396$^+$ p-tau (red) and **b** PHF-1$^+$ tau (red) and nuclei (DAPI, blue) in patients with DLB and FTLD. DLB, Dementia with Lewy bodies; FTLD, Frontotemporal lobar degeneration with frontotemporal dementia; ALS, Amyotrophic Lateral Sclerosis; PSP, progressive supranuclear palsy. F, female; M, male; age (in years).

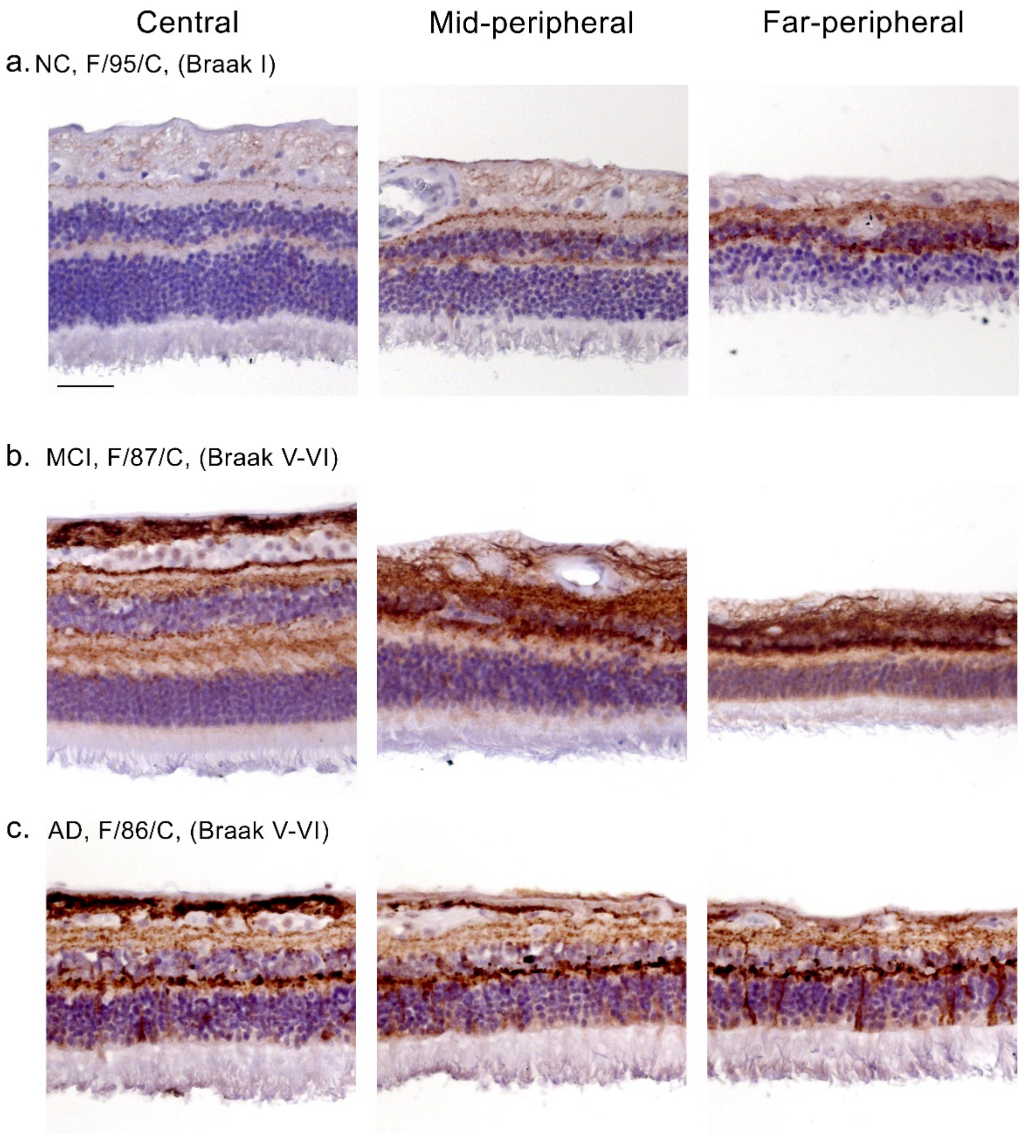


**Supplementary Figure 4.** Regional differences in retinal pS396-tau. **a-c.** Representative images of peroxidase-based staining of pS396$^{+}$p-tau on retinal cross-sections from MCI, AD patients and normal cognition (NC) controls grouped by central, mid-peripheral, and far-peripheral retinal regions.
